# Prevalence and associated burden of shift work disorder (SWD) in healthcare shift workers in the UK National Health Service (NHS): a cross-sectional survey

**DOI:** 10.1136/bmjopen-2026-118265

**Published:** 2026-11-05

**Authors:** Thavapriya Sugavanam, Vaughan Parsons, Xiaoxiao Ling, Anne Stagg, Ira Madan, Stavros Petrou, Rachel Sharman, Simon D Kyle, Leah Holmes, Annie Keane, Ly-Mee Yu, Forrest Tin Wai Cheung, David W Ray

**Affiliations:** 1 Oxford Centre for Diabetes, Endocrinology and Metabolism (OCDEM), Radcliffe Department of Medicine, University of Oxford, Oxford, UK; 2 John Radcliffe Hospital, NIHR Oxford Biomedical Research Centre, Oxford, UK; 3 NIHR Oxford Health Biomedical Research Centre, Oxford, UK; 4 Occupational Health, Safety and Wellbeing Service, Guy’s and St Thomas’ NHS Foundation Trust, London, UK; 5 School of Life Sciences and Medicine, King’s College London, London, UK; 6 London Centre for Work and Health, London, UK; 7 Nuffield Department of Primary Care Health Sciences, University of Oxford, Oxford, UK; 8 Guy's and St Thomas’ NHS Foundation Trust, London, UK; 9 Sir Jules Thorn Sleep and Circadian Neuroscience Institute, Nuffield Department of Clinical Neuroscience, University of Oxford, Oxford, UK; 10 Vocal, Manchester University NHS Foundation Trust, Manchester, UK

**Keywords:** SLEEP MEDICINE, OCCUPATIONAL & INDUSTRIAL MEDICINE, Quality of Life, Health Workforce, MENTAL HEALTH, Workplace

## Abstract

**Abstract:**

**Objectives:**

To determine the prevalence and associated burden of shift work disorder (SWD) in shift workers in the UK National Health Service (NHS).

**Design:**

Cross-sectional survey.

**Setting:**

Participants were recruited from two tertiary care NHS Foundation Trusts in England between April and June 2025.

**Participants:**

Those employed by the NHS who had worked non-standard hours (defined as work scheduled after 18:00 or before 07:00) for a minimum of 3 months.

**Primary and secondary outcome measures:**

Prevalence of SWD was assessed using a screening tool, the Shift Work Disorder Index (SWDI). The burden of SWD was evaluated using the EuroQol 5-Dimension 5-Level (EQ-5D-5L) and EQ Visual Analogue Scale (EQ-VAS) for health-related quality of life (HQoL), Work Productivity and Activity Impairment Questionnaire for work productivity, Patient Health Questionnaire-2 for depression and Generalised Anxiety Disorder-2 for anxiety symptoms.

**Results:**

Of 534 respondents, 458 (86%) were eligible and 443 (97%) completed the survey. Mean age was 36.1±10.7 years. The majority were female registered nurses, worked rotating shifts and were involved in shift work for 1–5 years.

Based on the SWDI, 71% (n=315/443; 95% CI 67% to 75%) met criteria for probable SWD. Probable SWD was significantly more prevalent among females; registered nurses and midwives; participants in higher NHS pay bands (band 5 and above); participants identifying as White; those working rotating shifts; those with more than 5 years of shift work; and those reporting a long-term health condition.

Participants with probable SWD had poorer HQoL (EQ-5D-5L: Z=−5.62, p<0.001, r=0.27; EQ-VAS: Z=−4.41, p<0.001, r=0.21), greater presenteeism (Z=−3.56, p<0.001, r=0.19), higher overall work impairment (Z=−3.44, p<0.001, r=0.19) and greater activity impairment (Z=−6.64, p<0.001, r=0.32) compared with participants without SWD.

Participants with probable SWD had more than a fourfold higher odds of possible depression (OR=4.35, 95% CI 1.97 to 9.61, p<0.001) and more than a threefold higher odds of possible anxiety (OR=3.52, 95% CI 1.52 to 8.12, p=0.003).

Most participants (n=284/315, 90%) reported trying at least one strategy to help their sleep, of which the most common were caffeine and naps. The majority (n=288/315, 91%) did not seek or receive support for their sleep difficulties.

**Conclusions:**

The prevalence of probable SWD appears to be high in NHS shift workers. This is associated with poorer HQoL, depression, anxiety and work productivity outcomes. Accessible, targeted, cost-effective, evidence-based interventions for SWD are required.

**STRENGTHS AND LIMITATIONS OF THIS STUDY**

- The adequately powered sample and minimal missing data are study strengths.
- The study sample closely reflects the National Health Service workforce, thereby supporting the generalisability and validity of the findings.
- The survey study design, use of convenience sampling and targeted recruitment of shift workers with sleep problems may have introduced selection bias.
- In addition, the cross-sectional design does not allow conclusions to be drawn about the directionality or causality of the observed associations.
- The Shift Work Disorder Index used to define shift work disorder (SWD) is a screening tool and hence a definite diagnosis of SWD could not be made.

## Introduction

Working non-standard hours (defined as work scheduled after 18:00 or before 7:00) is common in healthcare, transportation, hospitality and manufacturing sectors. This includes nights, early morning, late evening and rotating shifts. According to the 2022 Labour Force Survey, approximately 27% of the working population in the UK (ie, around 8.7 million people), were night-time workers.^1^ The National Health Service (NHS), which provides 24-hour care for patients, is the largest employer of shift workers in the UK. Approximately 12.5% of its 1.5 million staff work night shifts exclusively with many more working on rotating day-night shifts.^2^

Sleep problems are common among shift-working populations, and at the more severe end this is classified as shift work sleep disorder (SWD), a recognised sleep disorder by both the International Classification of Sleep Disorders (ICSD-3) and the Diagnostic and Statistical Manual of Mental Disorders (DSM-5).^3 4^ Diagnosis of SWD requires a complaint of insomnia and/or excessive sleepiness associated with a work schedule that overlaps with the usual sleep period, persisting for at least 3 months, resulting in functional impairment, and that is not better explained by another sleep, medical or mental disorder.^3–5^ The ICSD-3 recommends that the sleep-wake patterns are assessed using a 14-day sleep log or actigraphy to confirm a SWD diagnosis.^3^

Treatments for SWD are often directed at its core symptomology, including circadian misalignment, excessive sleepiness and sleep disturbances.^6^ Existing strategies encompass both individual-level (eg, sleep hygiene) and organisational-level (eg, shift rostering) approaches. Individual-level interventions may be pharmacological (eg, melatonin prescription) or non-pharmacological (eg, bright light therapy).^6–9^ However, the available evidence supporting these interventions remains limited, and no standardised treatment pathway has been established for SWD.^7–10^

Two systematic reviews have estimated the prevalence of SWD in recent years. The most recent review by Zhao *et al*, which focused specifically on nurses, included 24 studies and reported an estimated SWD prevalence of 45.5% (95% CI 39.2% to 51.8%).^11^ In contrast, the review by Pallesen *et al* included shift workers across multiple sectors and reported a lower estimated prevalence of 26.5% (95% CI 21.0% to 32.8%) based on 29 included studies.^12^ Both reviews highlighted substantial heterogeneity across the included studies (I^2^=98.5% in both) and wide variations in reported prevalence rates (0%–84%).^11 12^ However, none of the studies included in either review was conducted in the UK, and therefore, the prevalence of SWD in the UK remains unknown.

To effectively design and evaluate interventions for SWD within the NHS, it is essential to estimate the prevalence of SWD across the shift-working members of the healthcare workforce and evaluate the associated burden, the occupational and demographic groups in which interventional need is greatest, and the mitigating strategies used by those affected. Based on existing evidence,^11–13^ we hypothesised that probable SWD would be common among NHS staff working non-standard hours and expected it to be associated with adverse health and occupational outcomes. Therefore, the primary objectives of this study were to:

- Determine the prevalence of probable SWD in NHS staff who work non-standard hours (defined as work scheduled after 18:00 or before 07:00).
- Identify the associated health and economic burden of probable SWD in this population. This included health-related quality of life (HQoL), depression, anxiety, work productivity and activity limitation.

The secondary objective of this study was to describe strategies and support that shift-working NHS staff have used to help with their sleep, how effective these were, and the barriers (challenges/obstacles) to their use.

## Methods

### Study design and sampling

A cross-sectional survey design was adopted to address the above-stated aims. A non-probability volunteer (convenience) sampling strategy was followed. Based on an estimated prevalence rate of SWD of 20%,^12^ with a 95% CI ranging from 16.3% to 24.1% calculated using the Clopper-Pearson method, a total sample size of 424 participants was determined to be required.

### Study setting

Participants were recruited between April and June 2025 from two large NHS Foundation Trusts located in London and the East Midlands, England. Both sites were tertiary referral centres (acute hospital foundation trust settings). Study promotion was carried out by the on-site occupational health teams and communications teams using existing internal online channels, such as staff bulletins and health and well-being newsletters, as well as in-person distribution of promotional posters and flyers. As it was not possible to selectively target shift workers in the Trusts, the study was promoted to all NHS staff at the site, with study materials emphasising that the survey aimed to capture the experiences of shift workers regarding sleep and overall health.

### Study population

A participant could take part in the study if they fulfilled the following inclusion criteria:

1. Employed by the participating NHS Trusts (confirmed via a valid NHS email address).
2. Worked shifts that involved working non-standard hours (defined as work scheduled after 18:00 or before 07:00).
3. Worked non-standard hours for a minimum of 3 months.

Eligibility was confirmed at the start of the survey using three questions that aligned with the above inclusion criteria. Participation in the survey was not restricted by occupational roles. There were no other exclusion criteria for this study.

### Study procedures

The survey could be completed either online (Qualtrics, Provo, Utah, USA) or on paper (made available in staff communal areas) and took approximately 20 min to complete. Participants were asked to provide consent before proceeding with the survey. Participants were informed that they could withdraw from the study at any time during the survey, without providing a reason, by closing the browser or ceasing completion of the paper survey. Due to the anonymous nature of the study, participants were made aware that their responses could not be changed or withdrawn once the survey was completed and submitted. Participants were offered a £15 gift voucher on survey completion as a token of appreciation.

### Survey instrument

The survey included a battery of well-validated and widely used scales and bespoke questions to address the aims of the study.

The Shift Work Disorder Index (SWDI), a six-item self-report measure that assesses for symptoms of SWD as defined by the DSM-5, was used to determine SWD.^13^ The measure demonstrated excellent internal consistency (α=0.94) and good convergent and discriminant validity in a sample of nurses. Given its relevance to the assessment of SWD and its strong psychometric properties, the SWDI was selected for use in this study.^13^ The SWDI assesses the frequency and duration of shift work, and sleepiness, insomnia (difficulty falling or staying asleep), distress and social impairment associated with shift work; each on a 0 to 4 Likert scale with anchors varying according to the item. Participants were classified as having probable SWD if they met all of the following criteria: a score of ≥2 on the first SWDI item (indicating working non-standard hours at least 1–3 days per week); a score of ≥2 on either the second or third item (insomnia and/or sleepiness); a score of ≥2 on either the fourth or fifth item (distress and/or functional impairment); and a score of ≥3 on the sixth item (shift work duration >3 months), thereby meeting the minimum DSM-5 criteria for SWD.

HQoL was assessed using the EuroQol 5-Dimension 5-Level (EQ-5D-5L). The EQ-5D-5L contains a descriptive system that contains five dimensions (mobility, self-care, usual activity, pain/discomfort and anxiety/depression), each rated on five levels (no problems, slight problems, moderate problems, severe problems and extreme problems).^14 15^ The responses to the EQ-5D-5L were converted into utility scores using the mapping function currently recommended by the UK National Institute for Health and Care Excellence (NICE).^16 17^ Utility scores are indexed at 0 (equivalent to death) and 1 (perfect health) but can be negative for health states considered worse than death, with higher scores indicating better health. The EQ-5D-5L also contains a Visual Analogue Scale (EQ-VAS) that ranges from 0 to 100, on which participants rate their overall health status, with higher scores reflecting better health.^14 15^

The Work Productivity and Activity Impairment Questionnaire: Specific Health Problem V2.0 (WPAI) was used to evaluate impairments in work and activities.^18^ The WPAI assesses absenteeism (work time missed due to problem), presenteeism (impairment while working due to problem), overall work productivity loss and activity impairment attributable to a specific health condition (sleep problems in this study). WPAI outcomes were expressed as impairment percentages, with higher numbers indicating greater impairment and less productivity, that is, worse outcomes.^18^

The Patient Health Questionnaire-2 (PHQ-2)^19^ and the Generalised Anxiety Disorder-2 (GAD-2)^20^ were used to screen for depressive and anxiety symptoms, respectively. For both measures, total scores ranged from 0 to 6, with a score of ≥3 indicating possible depression or anxiety.

Other survey items comprised questions on demographics, occupational role, shift working patterns, presence of any long-term conditions (LTCs, defined as any physical or mental health condition lasting for 12 months or more), diagnosed sleep disorders, sleep duration and accidents or near misses due to sleep difficulties. Participants were also asked about the impact of working non-standard hours on eating habits (eg, food choices and/or meal patterns).^21 22^ Lastly, participants were asked about sleep strategies (eg, over-the-counter medication) and sleep support (provider and type, eg, general practitioner (GP)—sleep hygiene) they had tried or used, their perceived effectiveness and barriers encountered when attempting to address sleep difficulties (eg, lack of support).

### Data analysis

Data analysis was undertaken using SPSS (V.30.0; IBM) and R (V.4.3.2; R Foundation for Statistical Computing, Vienna, Austria). Duplicate survey responses identified via participants’ work email addresses were removed (n=8). Missing data were minimal, with 6.3% (n=28/443) of participants having missing values (27 respondents had missing data on one variable and one respondent had missing data on two variables); thus no missing data were imputed. Responses of ‘don’t know’ and ‘prefer not to answer’ were included in descriptive analyses but coded as missing and excluded listwise for inferential analyses. Some categorical variables (eg, ethnicity, occupational role) were collapsed into broader groups to facilitate analysis. To limit extreme responses for the number of hours worked and hours missed on the WPAI, a maximum value of 72 hours was applied.^23^ For participants who indicated that they did not work that week (ie, 0 hours worked), the subsequent work impairment item on the WPAI was not applicable, and any data provided for that item were considered invalid. For sleep duration, values below 3 hours per day were excluded as implausible, while all higher values were retained for analysis. Participants with missing data for a given variable were excluded only from analyses involving that variable.

The primary outcome measure for the study was the prevalence of the SWD, which was defined using the SWDI. The point prevalence of SWD was calculated using the number of participants with probable SWD as the numerator and the number of participants who completed the SWDI questionnaire as the denominator.

The distribution of variables was assessed for normality, and statistical tests were selected accordingly. Descriptive statistics—means (SD) or medians (IQRs) for continuous variables, and frequencies and percentages for categorical variables—were calculated to summarise participant characteristics. Differences between participants with and without probable SWD in demographic and survey outcome variables were evaluated using independent-samples t-tests or Mann-Whitney U tests for continuous variables and χ^2^ tests or Fisher’s exact tests for categorical variables. Effect sizes, reflecting between-group differences in distributions, were reported where applicable. A significance level of p<0.05 was applied.

The association between probable SWD and health and work outcomes was examined using multiple linear regression for continuous outcomes and logistic regression for binary outcomes. Probable SWD status was the primary explanatory variable, and the outcomes included EQ-5D-5L utility scores, EQ-VAS scores, WPAI absenteeism, presenteeism, overall work impairment, activity impairment, and likely depression and anxiety based on the PHQ-2 and GAD-2, respectively. Within the regression analyses, morning-shift workers (n=6) were combined with the reference category of rotational shift workers due to the small sample size. In model 1, analyses were adjusted for potential confounders such as site, age, sex, ethnicity, occupational role, presence of an LTC, and self-reported sleep duration. In model 2, shift-related variables such as shift type, shift-work duration, and employment type (full-time, part-time) were added to assess whether the associations between probable SWD and the outcomes were independent of these factors. Two sensitivity analyses were undertaken to assess structural uncertainty related to model choice for the skewed EQ-5D-5L utility scores, using ordinary least squares regression with bootstrapped standard errors and a beta regression model^24 25^ (see online supplemental material 1 for full details).

Results are reported in accordance with the Strengthening the Reporting of Observational Studies in Epidemiology (STROBE) guidelines (online supplemental material 2, S table 1).^26^

### Patient and public involvement

Two, 2-hour online meetings with preparation were held with an NHS Nightshift Workers Research Advisory Group (n=8, 5 female and 3 male) members, to help design this study. Group members worked within 7 NHS Trusts across England; in community settings, hospitals and NHS infrastructure and had intersectional diversity across types and seniority of roles, geographies and protected characteristics. The group provided input in choosing the study title, refining the content and structure of the survey and the participant-facing materials (eg, participant information sheet). The survey results were presented to the group in a follow-up online meeting to obtain their reflections, feedback and recommendations for future practice.

## Results

Due to the promotion and recruitment method used in the study, it was not possible to determine how many NHS staff had received information about the study, and thus the response denominator could not be established. Of the 534 participants who attempted the survey, 458 (86%) were eligible to take part, and of those eligible, 443 (97%) completed the survey (figure 1). Reasons for non-completion could not be explored due to the nature of the study design. Of the 443 eligible participants, 248 (54%) were from site 1 and 195 (43%) were from site 2.

**Figure 1:**
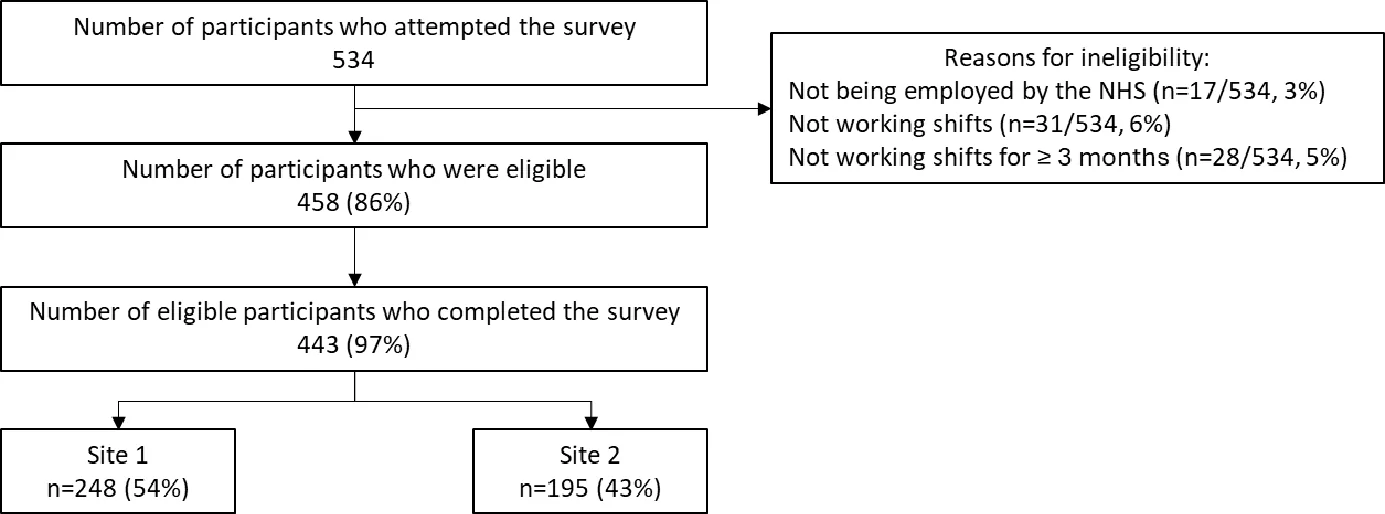
Participant recruitment and flow chart. NHS, National Health Service.

### Participant characteristics

The characteristics of the participants are presented in table 1. The mean age of the participants was 36.1±10.7 years (range: 19 to 71 years). The majority of the participants were female (n=366, 83%); registered nurses (n=218, 49%); worked on a full-time basis (n=320, 70%); were either employed on an NHS band 5 (n=120, 27%) or band 6 (n=135, 31%) level; worked rotating shifts (n=286, 65%); and involved in shift work for 1 to 5 years (n=196, 44%) (range: 3 months to 44 years). The ethnicity of the participants was mixed, with 44% (196) identifying as White, 32% (143) as Asian and 12% (54) as Black. Around 37% (n=165) of participants reported having a long-term physical or mental health condition, with 9% (n=41) reporting having three or more LTCs. Anxiety/panic disorder (n=60), arthritis or ongoing problem with back or joints (n=54) and depression (n=48) were the most reported LTCs. In total, 3% (n=14) of participants reported a diagnosis of sleep disorders such as insomnia and sleep-disordered breathing. The mean sleep duration was 6.2±1.3 hours per day (range: 3 to 10.5 hours). Most participants self-reported having sleep problems due to working shifts (n=363, 82%). Approximately 31% (n=135) reported having one or more accidents or near misses (motor vehicle, home or work related) over the last year due to sleepiness.

**Table 1:** Characteristics of participants

Variables		All participants	Participants with probable SWD	Participants without SWD	P value
Number of participants (%)		443	315 (71%)	128 (29%)	
Age in years (n=443) Mean±SD		36.09±10.71	36.01±10.47	36.30±11.30	0.79
Site (n=443)	Site 1	248 (56%)	178 (57%)	70 (55%)	0.73
	Site 2	195 (44%)	137 (43%)	58 (45%)	
Sex (n=443)	Female	366 (83%)	270 (86%)	96 (75%)	0.02*
	Male	74 (17%)	43 (14%)	31 (24%)	
	Non binary†	1	1	0	
	Prefer not to say†	2	1	1	
Employment type (n=443)	Full-time (37.5 or more hours/week)	320 (72%)	223 (71%)	97 (76%)	0.29
	Part-time (less than 37.5 hours/week)	123 (28%)	92 (29%)	31 (24%)	
NHS job band (n=443)	Band 2	25 (6%)	16 (5%)	9 (7%)	0.003*
	Band 3	61 (14%)	37 (12%)	24 (19%)	
	Band 4	27 (6%)	12 (4%)	15 (12%)	
	Band 5	120 (27%)	87 (28%)	33 (26%)	
	Band 6	135 (31%)	104 (33%)	31 (24%)	
	Band 7 and above	35 (8%)	31 (10%)	4 (3%)	
	Other	36 (8%)	26 (8%)	10 (8%)	
	Prefer not to say†	4 (1%)	2 (1%)	2 (2%)	
Occupational role‡ (n=443)	Medical/dental	39 (9%)	29 (9%)	10 (8%)	0.01*
	Registered nurse	218 (49%)	165 (52%)	53 (41%)	
	Midwives	43 (10%)	34 (11%)	9 (7%)	
	Nursing auxiliary/nursing assistant/healthcare assistant	45 (10%)	24 (8%)	21 (16%)	
	Admin and clerical	50 (11%)	28 (9%)	22 (17%)	
	Wider healthcare team (eg, maintenance, ambulance)	9 (2%)	8 (3%)	1 (1%)	
	Allied health professionals/healthcare scientists/scientific and technical	39 (9%)	27 (9%)	12 (9%)	
Ethnicity‡ (n=443)	White: English/Welsh/Scottish/Northern Irish/British/Irish/other	196 (44%)	153 (49%)	43 (34%)	0.03*
	Asian/Asian British	143 (32%)	91 (29%)	52 (41%)	
	Black/African/Caribbean/Black British	54 (12%)	37 (12%)	17 (13%)	
	Mixed/multiple ethnic group	19 (4%)	16 (5%)	3 (2%)	
	Other ethnic groups	18 (4%)	12 (4%)	6 (5%)	
	Prefer not to answer†	13 (3%)	6 (2%)	7 (6%)	
Type of shift work (n=443)	Evening/late shifts (work usually finished between 18:00 and midnight)	63 (14%)	45 (14%)	18 (14%)	<0.001*§
	Early morning shifts (work usually started before 7:00)	6 (1%)	0 (0%)	6 (5%)	
	Night shifts (work hours included at least 3 hours between 23:00 and 06:00)	88 (20%)	57 (18%)	31 (24%)	
	Rotating shifts (periodically changed or had irregular work hours)	286 (65%)	213 (68%)	73 (57%)	
Shift work duration (n=443)	Less than 1 year	35 (8%)	22 (7%)	13 (10%)	0.003*
	1–5 years	196 (44%)	124 (39%)	72 (56%)	
	6–10 years	101 (23%)	84 (27%)	17 (13%)	
	11–20 years	78 (18%)	58 (18%)	20 (16%)	
	More than 20 years	33 (7%)	27 (9%)	6 (5%)	
Self-report of sleep problems and duration (n=443)	I don’t have a problem	80 (18%)	30 (10%)	50 (39%)	<0.001*
	Less than 1 month	22 (5%)	11 (4%)	11 (9%)	
	1–2 months	24 (5%)	13 (4%)	11 (9%)	
	3–6 months	46 (10%)	32 (10%)	14 (11%)	
	7–12 months	30 (7%)	25 (8%)	5 (4%)	
	More than 1 year	241 (54%)	204 (65%)	37 (29%)	
Accidents/near misses (motor vehicle, home or work related) due to sleepiness (n=443)	0 accidents	244 (55%)	148 (47%)	96 (75%)	<0.001*§
	1–5 accidents	121 (27%)	97 (31%)	24 (19%)	
	6–10 accidents	7 (2%)	5 (2%)	2 (2%)	
	More than 10 accidents	7 (2%)	7 (2%)	0	
	Do not know	53 (12%)	49 (16%)	4 (3%)	
	Prefer not to answer	11 (3%)	9 (3%)	2 (2%)	
Sleep duration (self-reported average per day over the last month) (n=432) Mean±SD		6.2±1.2	6.2±1.2	6.3±1.1	0.39
Presence of long-term health condition (n=443)	Yes	165 (37%)	132 (42%)	33 (26%)	0.003*
	No	258 (58%)	172 (55%)	86 (67%)	
	Prefer not to answer†	20 (5%)	11 (4%)	9 (7%)	
Impact of shift work on eating habits (n=443)	No impact	40 (9%)	11 (4%)	29 (23%)	<0.001*
	Slight impact	98 (22%)	57 (18%)	41 (32%)	
	Moderate impact	182 (41%)	141 (45%)	41 (32%)	
	Extreme impact	123 (28%)	106 (34%)	17 (13%)	

All percentages presented are based on the within group value for that particular variable.

* Statistically significant difference (p<0.05).

† Not included in the statistical analysis.

‡ Categories have been collapsed to broader top-level groups for ease of reporting.

§ Fisher’s exact p value.

NHS, National Health Service; SWD, shift work disorder.

Participant characteristics differed significantly between sites in terms of ethnicity, employment type, occupational role, pay bands and shift work patterns (see online supplemental material 3, S table 2). Site 1 had a higher proportion of participants in higher pay bands (band 6 and above), in midwifery roles, working evening/late shifts and identifying as ‘black’. Site 2 had more nursing assistants, participants working night shifts and a higher proportion identifying as ‘white’.

### SWD prevalence

Based on the SWDI, 71% (n=315) (95% CI 67% to 75%) met the criteria for probable SWD. Of participants grouped as probable SWD, the majority (n=277, 88%) reported experiencing both insomnia and sleepiness. Similarly, the majority of participants (n=235, 75%) endorsed both distress and impairment as a consequence of working non-standard hours.

### Differences in participant characteristics based on probable SWD status

Statistically significant differences in participant characteristics were observed between participants with and without probable SWD (table 1). Probable SWD was significantly more prevalent among females; registered nurses and midwives; participants in higher NHS pay bands (band 5 and above); participants identifying as White; those working rotating shifts; those with more than 5 years of shift work; and those reporting an LTC, and was less prevalent among nursing assistants, administrative/clerical staff and participants identifying as Asian/Asian British. No significant differences were observed by site, age, employment type or self-reported sleep duration. Of the 135 participants who reported having accidents/near misses over the past year due to working shifts, 109 (81%) met the criteria for probable SWD.

### Health-related quality of life

Participants with probable SWD had lower EQ-5D utility and VAS scores than those without, indicating poorer HRQoL in the probable SWD group. Between-group differences were statistically significant for both the EQ-5D utility (Z=−5.62, p<0.001) and VAS scores (Z=−4.41, p<0.001) (table 2). Effect sizes were small to moderate (EQ-5D: r=0.27; VAS: r=0.21).

**Table 2:** Main study outcomes by probable shift work disorder status

Variables		All participants (n=443)	Participants with probable SWD (n=315)	Participants without SWD (n=128)	P value	Effect size*
EQ-5D Utility score	Sample size n (%)	440 (99%)	313	127	<0.001†	0.27
	Median (IQR)	0.82 (0.18)	0.80 (0.21)	0.89 (0.19)		
EQ VAS score	Sample size n (%)	443 (100%)	315	128	<0.001†	0.21
	Median (IQR)	75 (25)	74 (25)	80 (20)		
WPAI Absenteeism	Sample size n (%)	374 (85%)	271	103	0.53	–
	Median (IQR)	0% (0.9%)	0% (0%)	0% (5.1%)		
WPAI Presenteeism	Sample size n (%)	343 (77%)	254	89	<0.001†	0.19
	Median (IQR)	40% (40%)	40% (40%)	20% (45%)		
WPAI Overall work impairment	Sample size n (%)	342 (77%)	253	89	<0.001†	0.19
	Median (IQR)	40% (40%)	40% (30%)	25% (50%)		
WPAI Activity impairment	Sample size n (%)	443 (11%)	315	128	<0.001†	0.32
	Median (IQR)	50% (40%)	50% (30%)	30% (50%)		
PHQ-2	Likely to be depressed	103 (23%)	88 (28%)	15 (12%)	<0.001†	0.17
	Not likely to be depressed	340 (77%)	227 (72%)	113 (88%)		
	Mean±SD	1.8±1.5	2.1±1.6	1.2±1.2	–	–
GAD-2	Likely to be anxious	93 (21%)	80 (25%)	13 (10%)	<0.001†	0.17
	Not likely to be anxious	350 (79%)	235 (75%)	115 (90%)		
	Mean±SD	1.7±1.6	2.0±1.7	1.1±1.4	–	–

* Effect size reflects the between-group difference in distributions.

† Statistically significant difference.

EQ-5D, EuroQol Five-Dimensions; GAD-2, Generalised Anxiety Disorder-2; PHQ-2, Patient Health Questionnaire-2; SWD, shift work disorder; VAS, Visual Analogue Scale; WPAI, Work Productivity and Activity Impairment.

These differences remained statistically significant in regression analysis, with probable SWD independently associated with lower EQ-5D-5L utility (adjusted R²=0.29) and VAS scores (adjusted R²=0.18) in both models (all p<0.001; n=400) (online supplemental material 4, S table 3–6). Sensitivity analyses using alternative model specifications yielded consistent results for the probable SWD group (online supplemental material 5, S tables 17–20).

### Work productivity and activity limitation

Valid data were available for the WPAI domains as follows: absenteeism (n=374, 85%), presenteeism (n=343, 77%), overall work impairment (n=342, 77%) and activity impairment (n=443, 100%). Reductions in sample size for the first three domains were due to missing or invalid responses, including inapplicability of the work impairment item for participants reporting no work that week. All WPAI domains, except absenteeism, showed statistically significant differences between participants with and without probable SWD. Participants with probable SWD symptoms reported greater presenteeism (Z=−3.56, p<0.001, r=0.19), higher overall work impairment (Z=−3.44, p<0.001, r=0.19) and greater activity impairment (Z=−6.64, p<0.001, r=0.32) due to their sleep problems, indicating small to moderate effect sizes (table 2).

Multivariable regression analyses confirmed that these associations remained significant in both models for the three WPAI domains: presenteeism (n=312; model 1: adjusted R^2^=0.07, p<0.001; model 2: adjusted R^2^=0.07, p=0.002); overall work impairment (n=311; model 1: adjusted R^2^=0.06, p=0.003; model 2: adjusted R^2^=0.06, p=0.01); activity impairment (n=400; model 1: adjusted R^2^=0.17, p<0.001; model 2: adjusted R^2^=0.17, p<0.001). See online supplemental material 4, S tables 7–12 for details.

### Depression

A significantly higher proportion of participants in the probable SWD group met criteria for possible depression on the PHQ-2 (n=88, 28%) compared with the no SWD group (n=15, 21%). This between-group difference was statistically significant (x^2^=13.4, p<0.001) with a small effect size (φ=0.17) (table 2).

Regression analysis showed that participants with probable SWD had more than threefold higher odds of likely depression (OR=3.63, 95% CI 1.68 to 7.84, p=0.001) after adjustment for demographic variables (model 1) and fourfold higher odds (OR=4.35, 95% CI 1.97 to 9.61, p<0.001) after including the shift-related variables in the analysis (model 2) (see online supplemental material 4, S tables 13 and 14, for details).

### Anxiety

A significantly higher proportion of participants in the probable SWD group met criteria for possible anxiety based on the GAD-2 (n=80, 25%) compared with the no SWD group (n=13, 10%). This between-group difference was statistically significant (x^2^=12.8, p<0.001) with a small effect size (φ=0.17) (table 2).

Regression analysis showed that participants with probable SWD had more than threefold higher odds of likely anxiety (OR=3.48, 95% CI 1.53 to 7.94, p=0.003) after adjustment for demographic variables (model 1) and similar odds (OR=3.52, 95% CI 1.52 to 8.12, p=0.003) after including the shift-related variables in the analysis (model 2) (see online supplemental material 4, S tables 15 and 16 for details).

### Impact on eating habits (eg, food choices and/or meal patterns)

A significantly higher proportion of participants in the probable SWD group reported a moderate to extreme impact of shift work on eating habits compared with the non SWD group (x^2^=62.2, p<0.001) (table 1).

When asked to expand on their answers in free-text responses, participants in the probable SWD group reported that shift work and disrupted sleep led to irregular eating patterns. Fatigue, long shifts, missed or poorly timed breaks and limited time to cook often led to reliance on convenience or processed foods. Participants also described fluctuating appetite, often linked to weight changes and digestive issues, while a small number reported using coping strategies to manage these challenges.

### Sleep strategies and support

Most participants in the probable SWD group (n=284/315, 90%) reported trying at least one strategy to help with their sleep and/or to reduce sleepiness at work (table 3). Participants could select any number of strategies from the provided list. The most common strategies tried were caffeine (incl. coffee, tea and energy drinks) (n=164/315, 52%), naps (including at home and at work) (n=150/315, 48%), changes in diet (incl., quality, quantity and timing of meals and snacks) (n=129/315, 41%), change in physical activity and exercise (n=115/315, 37%) and over-the-counter sleeping aids (eg, herbal teas, supplements or non-prescription sleeping tablets) (n=103/315, 33%). Within the ‘other’ strategies, eight participants (3%) had indicated that they take prescribed sleep medications (eg, Melatonin, Zopiclone).

**Table 3:** Strategies tried to help to sleep and/or reduce sleepiness at work

Strategies tried to help to sleep and/or reduce sleepiness at work	No. of participants* (n=315)	Extremely effective	Very effective	Moderately effective	Slightly effective	Not effective at all
Caffeine (incl. coffee, tea and energy drinks)	164 (52%)	4 (2%)	19 (12%)	57 (35%)	71 (43%)	13 (8%)
Naps (incl. at home and at work)	150 (48%)	10 (7%)	15 (10%)	46 (31%)	56 (37%)	23 (15%)
Changes in diet (including, quality, quantity and timing of meals and snacks)	129 (41%)	–	16 (12%)	32 (25%)	53 (41%)	27 (21%)
Change in physical activity and exercise	115 (37%)	5 (4%)	14 (12%)	35 (30%)	44 (38%)	17 (15%)
Over the counter sleeping aids (eg, herbal teas, supplements or non-prescription sleeping tablets)	103 (33%)	–	7 (7%)	23 (22%)	58 (56%)	15 (15%)
Changes to light exposure	104 (33%)	8 (8%)	15 (14%)	42 (40%)	29 (28%)	10 (10%)
Use of mobile phone apps (incl. apps that support sleep)	60 (19%)	1 (2%)	3 (5%)	11 (18%)	28 (47%)	17 (28%)
Sleep-tracking device (eg, using a wristband or smartphone app)	59 (19%)	1 (2%)	–	13 (22%)	19 (32%)	24 (41%)
Self-help psychological therapies	44 (14%)	3 (7%)	3 (7%)	16 (36%)	15 (34%)	6 (14%)
Alcohol	33 (11%)	1 (3%)	–	10 (30%)	12 (36%)	10 (30%)
Others†	32 (10%)	6 (19%)	5 (16%)	9 (28%)	8 (25%)	2 (6%)
Other illicit substance(s)	3 (1%)	–	–	2 (67%)	1 (33%)	–

* Participants could choose more than one strategy and may be included in multiple rows.

† Strategies listed under ‘others’ included lifestyle and behavioural approaches (eg, sleep hygiene routines, listening to sleep tracks), environmental modifications (eg, sleep masks) and use of prescribed sleep medications (eg, melatonin, zopiclone).

In terms of the effectiveness of these interventions, only a small percentage of participants (<20%) rated any one intervention as ‘very effective’ or ‘extremely effective’. The majority of the participants indicated that the individual interventions were either slightly or moderately effective.

The majority of the participants (n=288/315, 91%) in the probable SWD group reported that they did not receive any support to help them sleep or to reduce sleepiness when working non-standard hours. Support from the GP (n=11/21, 52%) and manager at work (n=6/21, 29%) were the most commonly reported support among the few participants who indicated they received some support (n=21/315). The commonly reported types of support received from these professionals were prescription for sleep medication (n=8), sleep hygiene advice (n=7) and referral to cognitive behavioural therapy (CBT) (or any other similar psychological intervention) (n=7). Due to the small sample, the effectiveness of the support could not be meaningfully synthesised but was mostly distributed between slightly and moderately effective.

Busy schedule/time constraints (n=136/315, 43%), long waiting times for appointments (n=126/315, 40%) and lack of awareness about available sources of support (n=120/315, 38%) were the most commonly chosen factors that prevented participants from seeking support for their sleep problems (table 4).

**Table 4:** Factors that impede participants from seeking support

Factors that impede participants from seeking support	No. of participants† (n=315)
Lack of awareness about available sources of support	120 (38%)
Busy schedule/time constraints	136 (43%)
Long waiting times for appointments	126 (40%)
Lack of financial resources	94 (30%)
Don’t believe the problem is serious enough	74 (24%)
Low motivation	85 (27%)
Lack of support from work environment	88 (28%)
Fear of judgement from others (stigma and discrimination)	73 (23%)
Lack of access to healthcare support	69 (22%)
Potential side effects	31 (10%)
Fear of receiving a health diagnosis	29 (9%)
Mental health concerns	26 (8%)
Lack of support from family and friends	18 (6%)
Previous negative experiences	17 (5%)
Others*	15 (5%)
Lack of transportation	6 (2%)
Cultural or religious beliefs	4 (1%)
Language barriers	1 (0.3%)

* Barriers listed under ‘others’ included night shift culture normalising tiredness, nature of shift work and not being listened to by the GP.

† Participants could choose more than one strategy and may be included in multiple rows.

GP, general practitioner.

### Patient and public involvement feedback

The Research Advisory Group confirmed that the findings reflected their experiences, including the high prevalence of SWD. They broadly agreed with the results, although one member expected nap use to be more common. The group acknowledged that shift work–related sleep problems could increase the risk of accidents and felt this was exacerbated by staffing reductions within NHS settings. They also highlighted the lack of awareness of SWD among shift workers and managers, with some people being labelled as suffering with mental health issues. There was a strong consensus that support for SWD should be a priority.

## Discussion

This cross-sectional study of healthcare shift workers in the UK aimed to estimate the prevalence SWD, assess its burden in this population, and identify sleep strategies and support used by participants. The prevalence of probable SWD was estimated at 71% among the 443 participants who completed the survey. The presence of probable SWD was associated with poorer HRQoL, work productivity, depression and anxiety outcomes. Participants reported using several strategies such as caffeine and naps to manage their sleep difficulties, although these were perceived as only mildly effective. Professional help was very rarely sought.

### Comparison with other studies

#### SWD prevalence

The 71% prevalence of probable SWD identified in this study is much higher than the 45.5% and the 26.5% estimated in the recent systematic reviews of SWD prevalence.^11 12^ Although there was some overlap in the included studies within these reviews, the prevalence across the included studies in Zhao *et al* review ranged between 16% and 84%,^11^ while the prevalence range was between 0 and 84%in the Pallesen *et al* review,^12^ highlighting the broad variation in SWD prevalence estimation. Another smaller review of five studies showed a similar wide range of insomnia prevalence (13% to 77%) in shift workers.^27^ The included studies in these reviews used a range of scale- or questionnaire-based tools (eg, Pittsburgh Sleep Quality Index) or applied the ICSD-2 or ICSD-3 criteria to determine SWD and/or insomnia, which likely contributed to variability in prevalence estimates.^6 12^ None of the above studies, excluding the study by the SWDI developers,^13^ used the SWDI that was employed in the current study to define SWD. The strength of the SWDI lies in its coverage of each element of the DSM-5 definition of SWD, thereby enhancing the validity and confidence of the screening and SWD classification.^13^ Nevertheless, it should be acknowledged that this is a screening estimate and not a definite diagnosis. A possible explanation for the high prevalence is selection bias, as although the study was promoted to all shift workers, its focus on sleep may have preferentially attracted individuals experiencing sleep problems.

#### SWD burden

Demographic characteristics relating to the female sex, white ethnic background, nursing roles and higher NHS pay bands and shift work characteristics such as longer duration of shift work and rotating shifts were observed to have a higher proportion of probable SWD. The association between longer duration of shift working and SWD, sleep and overall health is well documented.^28 29^ A recent study that analysed the UK BioBank dataset concluded that ‘Asian or Asian British’ or ‘Black or Black British’ (compared with being ‘White’) and the male sex were associated with lower odds for SWD,^30^ which was also observed in this study. The higher prevalence of probable SWD among females may partly relate to an earlier circadian phase and a greater tendency to report sleep-related problems, while observed ethnic differences may reflect response bias as well as genetic ancestry-related variations in sleep and circadian biology.^31 32^ Recent statistics show that of the 1.5 million staff employed by the NHS, more than three-quarters (76%) are female and around 70% identify themselves as ‘White’.^33–35^ As this group is at increased risk of developing SWD, it underscores the urgent need for effective strategies to detect, mitigate and treat SWD in the NHS.

The associations observed between probable SWD and outcomes of health and work in this study are supported by several studies globally.^13 36–40^ Over the past few decades, these outcomes have been proposed as confounders, predictors and mediators of SWD in multiple studies.^36–41^ The presence of an LTC was not only associated with probable SWD but was also a significant contributing variable in most of the regression models. Previous studies have established links between shift working and the development of several LTCs.^42–44^ Additionally, accidents and other safety issues have been discussed in relation to shift work^45 46^ and in this study the probable SWD group was more likely to report accidents. However, as accidents at work and personal life were combined into a single survey item, it was not possible to examine these associations separately.

#### Sleep strategies and support

The main strategies tried by shift workers to address their sleep problems were of a self-directed nature, in particular the use of caffeine and naps. This is of no surprise as there are no established guidelines available for the management of SWD. In the UK, NICE only provides some advice on the management of SWD (eg, sleep hygiene) and does not recommend one particular treatment.^47^ Recent reviews have attempted to consolidate existing evidence on sleep interventions in shift workers and indicate that there is an emerging body of potentially promising non-pharmacological interventions but caution on the low quality and quantity of the evidence.^7–9^ Results from this survey indicate that the majority of participants identified as having probable SWD also experienced insomnia symptoms, suggesting that the first-line treatment for insomnia, CBT for insomnia (CBTi), may represent an avenue of promise. However, standard CBTi may not be directly applicable to the shift-working population due to the circadian misalignment in this group.^48 49^ Consequently, there has been increased focus on adapting CBTi for shift workers.^48 50–55^ The effectiveness of these modified interventions is beginning to emerge and do show potential, particularly in the healthcare sector.^48 50–55^

It is also concerning that most participants in this study did not seek or receive support for their sleep problems. Several qualitative works have shown that shift workers tend to accept some of the issues of shift working as inevitable and have a tendency to not seek support.^56–58^ This underscores the importance of not only developing effective interventions for SWD but also ensuring they are accessible and acceptable to the shift workers.

### Implications for research, policy and practice

Although the study was conducted in an NHS healthcare setting, the findings may be generalisable to other sectors that employ shift workers (eg, transportation, emergency services, aviation), where similar work patterns and sleep challenges occur. The study findings emphasise the pressing need to support shift workers and manage SWD when it presents. Additionally, clear treatment guidelines and structured implementation strategies will be essential to ensure these interventions are translated into routine practice and reach the target population. For these implementation plans to be successful, organisations employing shift workers will have to play a crucial role in the prevention, support, and management of SWD. In practice, this may include integrating SWD screening into occupational health assessments, educating shift workers on recognising symptoms and healthy sleep behaviours, and training managers to support early identification and referral.^55 59^ Clear referral pathways will also be needed to facilitate timely diagnosis and treatment. Preventive strategies such as screening and managing sleep disorders before commencing shift work are now beginning to be considered to alleviate the impact of SWD.^60^ However, as indicated by the heterogeneity of reported SWD prevalence, future efforts should focus on developing consistent measures to identify workers who may be at risk of developing SWD. Alongside this, effective prevention, treatment and implementation strategies supported by coordinated individual and organisational engagement will be essential to reduce the burden of SWD.

### Strengths and limitations

This is the first study to produce probable SWD prevalence estimates for the UK healthcare sector. The adequately powered sample and minimal missing data are study strengths. The study sample closely reflects the NHS workforce, thereby supporting the generalisability and validity of the findings. However, the survey study design, use of convenience sampling and targeted recruitment of shift workers with sleep problems may have introduced selection bias. In addition, the cross-sectional design does not allow conclusions to be drawn about the directionality or causality of the observed associations. The SWDI is a validated screening instrument rather than a diagnostic tool; therefore, this study identified probable rather than clinically confirmed SWD. Similarly, the PHQ-2 and GAD-2 are screening tools and do not establish definitive diagnoses of depression and anxiety respectively. Hence, the possibility of misclassification should be considered, and the prevalence estimates and observed associations interpreted with caution. Objective measurements of sleep, alongside structured clinical interviews with shift workers, can lead to a more confident diagnosis of SWD but are resource-intensive and may introduce selection bias.

## Conclusions

This study identified an unexpectedly high prevalence of probable SWD among healthcare shift workers, with 71% of surveyed shift workers meeting the criteria for probable SWD. Probable SWD was associated with poorer HRQoL, depression, anxiety and work productivity outcomes. These findings highlight the substantial burden associated with probable SWD and support the need for accessible, targeted and evidence-based interventions to address SWD among healthcare shift workers.

## Supplementary material

10.1136/bmjopen-2026-118265 online supplemental file 1

## Data Availability

Data are available on reasonable request.
