# Supplementary material for: Prevalence and associated burden of shift work disorder (SWD) in healthcare shift workers in the UK National Health Service (NHS): a cross-sectional survey
Source: BMJ Open. 2026 Nov 5;16(11):e118265. doi: 10.1136/bmjopen-2026-118265 (PMC13756277; doi:10.1136/bmjopen-2026-118265)
Supplement: Supplementary file 1 [file bmjopen-2026-118265.reviewer_comments.pdf]

PEER REVIEW HISTORY

BMJ Open publishes all reviews undertaken for accepted manuscripts. Reviewers are asked to complete a checklist review form and are provided with free text boxes to elaborate on their assessment. These free text comments are reproduced below.

ARTICLE DETAILS

Title (Provisional)

Prevalence and associated burden of shift work disorder (SWD) in healthcare shift workers in the UK National Health Service (NHS)- A cross-sectional survey

Authors

Sugavanam, Thavapriya; Parsons, Vaughan; Ling, Xiaoxiao; Stagg, Anne; Madan, Ira; Petrou, Stavros; Sharman, Rachel; Kyle, Simon D; Holmes, Leah; Keane, Annie; Yu, Ly-Mee; Cheung, Forrest Tin Wai; Ray, , David W.

VERSION 1 - REVIEW

**Reviewer**	**1**
**Name**	**Dopelt, Keren**
**Affiliation**	**Ben-Gurion University of the Negev, Health Policy and Management**
**Date**	**20-Mar-2026**
**COI**	**None**

This is a well-conducted and clearly reported cross-sectional study addressing an important and clinically relevant topic, shift work disorder (SWD) among healthcare workers. There are several points that require clarification and more cautious interpretation:

1) The study objectives are clearly stated; however, explicit research hypotheses are not articulated.

2) The reported prevalence of probable SWD (71%) is substantially higher than estimates reported in previous literature. While this is an important finding, it raises concerns regarding potential selection bias, particularly given the use of convenience sampling and the broad recruitment strategy. This issue should be more explicitly acknowledged and discussed, including its implications for generalisability.

3) The measurement tools are well described; however, additional discussion of their validity and reliability, particularly in relation to the SWDI, would strengthen the methodological transparency.

4) SWD was defined using the SWDI, which is a screening instrument rather than a diagnostic tool. Although this is noted in the manuscript, the implications should be more strongly emphasised throughout, particularly in the abstract and discussion. The term “probable SWD” should be used consistently and interpreted with appropriate caution.

5) Given the cross-sectional design, causal inferences cannot be made. Some statements in the discussion and conclusions appear stronger than warranted by the study design. I recommend revising the wording to ensure that associations are not interpreted as causal relationships.

6) Consider briefly expanding on the generalisability of the findings beyond the NHS context.

7) The manuscript provides general practical implications; however, these remain broad and could be further developed to enhance applicability.

**Reviewer**	**2**
**Name**	**Fort, Emmanuel**
**Affiliation**	**University of Lyon, University of Lyon 1, University of Gustave Eiffel, UMRESTTE, UMR T_9405, F- 69373, LYON, France**
**Date**	**22-Jun-2026**
**COI**	**None**

Introduction:

Working non-standard hours (defined as work scheduled after 6 pm or before 7 am)

Working non-standard hours has several definitions according to countries and scientific literature. For exemple, In France, The French National Institute of Statistics and Economic Studies (INSEE) gives the definition : ”Non-standard working hours include Saturdays work, Sundays work, evenings work (from 8pm to midnight) or nights work (from midnight to 5am) at least one time in the last four weeks before the survey” for a Non-standard working hours. For the European Agency for Safety and Health at work, the standard working hours is between 0800 and 1800 on weekdays. On the opposite, it is the Non-standard working hours. Moreover, Organizations such as the U.S. Bureau of Labor Statistics and researchers studying work schedules typically include the following as non-standard hours:

- **Evening shifts** (e.g., work ending late at night)
- **Night shifts** or overnight work
- **Rotating shifts** (alternating between day, evening, or night schedules)
- **Split shifts** (two or more separate work periods in a day)
- **Weekend work** as a regular part of the schedule
- **Irregular or on-call schedules** where hours vary and are not fixed

Please justify the definition of Working non-standard hours you used.

Study design and sampling

How did authors select the prevalence rate of SWD of 20% for the calculation of the sample size?

Statistical analyses

Please add the statistical tests performed in order the approximative sentence “using appropriate statistical tests”. In table 1, only the Fisher test is mentioned, but the comparison of Sleep duration (self-reported average per day over the last month) according to probably SWD must have been performed with a comparative quantitative test. Please add this information in the statistical part in methods.

The use of a cut-off at 2 and more for the Patient Health Questionnaire-2 in order to identify the likelihood of depression is not a good choice. Indeed, this cut-off maximizes sensitivity (detecting more potential cases), at the cost of a higher number of false positives. Authors have presented the cut-off at 3 and more. This cut-off must be used in the following of the manuscript and in the analyses.

Same comment for the estimation of the prevalence of anxiety, the cut-off 3 and more must be used in the analyses.

For the UK EQ-5D-5L utility calculation, I thought that the correct reference was

Devlin NJ, Shah KK, Feng Y, Mulhern B, van Hout B. Valuing health-related quality of life: An EQ-5D-5L value set for England. *Health Economics*. 2018;27:7–22. https://doi.org/10.1002/hec.3564

Results

It is not clear if the analyse of the Accidents/Near misses (motor vehicle, home or work related) due to sleepiness in the last 12 months has been performed on the sub population of participants who has Shift work duration more than 1 year. I don’t think it is case regarding the number of total participants (n=443) in the table 1 for these 2 variables.

P16, ***Impact on eating habits (e.g., food choices and/or meal patterns)***

This paragraph is written like a discussion with an interpretation of the results but without references. There are a lot of results presented, please keep concise paragraph for dimension studied.

Discussion

The presence of probable SWD was associated with poorer HRQoL, work productivity, and mental health. Why author add poorer for health quality of life, and stay general for work productivity and mental health?

Moreover, mental health is not relevant short resume of the depression and anxiety probable issue. Indeed, mental health refers to a person's emotional, psychological, and social well-being, not only depressive symptoms and anxiety symptoms. Some of these dimensions were not evaluated in this study.

This study selected participants who worked shifts that involved working non-standard hours (defined as work scheduled after 6 pm or before 7 am and who worked non-standard hours for a minimum of 3 months.

Comparisons of prevalence of WSD with the 2 meta-analysis is quite difficult like these meta-analyses included several studies with several different definition of shift work. The criteria of selection if this study seems to be the more important and the more selective too. In this way, it is not surprising to find a higher prevalence of WSD. Moreover, the studies population in one of the meta-analyse included different type of workers, like this study focused on nurses.

The meta-analyse among nurses found a prevalence around 45%, with a lot of heterogeneity in shift works definition and also in the questionnaire used to evaluate the WSD.

VERSION 1 - AUTHOR RESPONSE

	Reviewer comments	Author response
**1**	**Reviewer 1**	
1.1	This is a well-conducted and clearly reported cross-sectional study addressing an important and clinically relevant topic, shift work disorder (SWD) among healthcare workers.	Thank you.
1.2	The study objectives are clearly stated; however, explicit research hypotheses are not articulated.	We have now added the hypotheses before the objectives.<br>*Based on existing evidence, we hypothesised that probable SWD would be common among NHS staff working non-standard hours and expected it to be associated with adverse health and occupational outcomes.* (p.8)
1.3	The reported prevalence of probable SWD (71%) is substantially higher than estimates reported in previous literature. While this is an important finding, it raises concerns regarding potential selection bias, particularly given the use of convenience sampling	We agree with the reviewer, and have already acknowledged these points in the discussion. Under SWD prevalence (p.24), we have explicitly stated:<br>*A possible explanation for the high prevalence is selection bias, as although the study was promoted to all shift workers, its focus on sleep*

	and the broad recruitment strategy. This issue should be more explicitly acknowledged and discussed, including its implications for generalisability.	*may have preferentially attracted individuals experiencing sleep problems*. (p.24)<br><br>Under limitations, we have mentioned:<br>*The study sample closely reflects the NHS workforce, thereby supporting the generalisability and validity of the findings. However, the survey study design, use of convenience sampling and targeted recruitment of shift workers with sleep problems may have introduced selection bias.* (p.26)<br><br>We respectfully believe that these statements adequately address the reviewer's concern, and therefore have not made further revisions to this section. However, we would be happy to expand the discussion further if the reviewer feels that additional clarification would be helpful.
1.4	The measurement tools are well described; however, additional discussion of their validity and reliability, particularly in relation to the SWDI, would strengthen the methodological transparency.	The sentence at the start of this section has been modified to emphasise that the most measures used in the survey are well established and validated.<br>*The survey included a battery of well-validated and widely used scales and bespoke questions to address the aims of the study. (p.9)*<br><br>The sentence on the psychometric properties of the SWDI has been expanded.<br>*The measure demonstrated excellent internal consistency (α=0.94) and good convergent and discriminant validity in a sample of nurses. Given its relevance to the assessment of SWD and its strong psychometric properties, the SWDI was selected for use in this study. (p.9)*<br><br>References are provided for each measure that document its psychometric properties. As these are well-established, widely used, and validated instruments, and given the manuscript's length, their psychometric properties are not described in detail.
1.5	SWD was defined using the SWDI, which is a screening instrument rather than a diagnostic tool. Although this is	We have now added that SWDI is a screening tool in the abstract.

	noted in the manuscript, the implications should be more strongly emphasised throughout, particularly in the abstract and discussion.	*Prevalence of SWD was assessed using a screening tool, the Shift Work Disorder Index (SWDI).* (p.4)<br><br>We have also added the following sentence to the discussion section of SWD prevalence.<br>*Nevertheless, it should be acknowledged that this is screening estimate and not a definite diagnosis.* (p. 24)<br><br>In addition, we have modified the sentence in the limitations section to acknowledge the implications of using a screening tool.<br>*The SWDI is a validated screening instrument rather than a diagnostic tool; therefore, this study identified probable rather than clinically confirmed SWD. Hence, the possibility of misclassification should be considered, and the prevalence estimates and observed associations interpreted with caution.* (p.26)
1.6	The term "probable SWD" should be used consistently and interpreted with appropriate caution.	We have double checked the manuscript to ensure that 'probable SWD' is used consistently in relation to the study procedures, findings and discussion. The term 'SWD' is only used when discussing other literature or SWD in general.
1.7	Given the cross-sectional design, causal inferences cannot be made. Some statements in the discussion and conclusions appear stronger than warranted by the study design. I recommend revising the wording to ensure that associations are not interpreted as causal relationships.	This sentence in the discussion section was modified to emphasise that these are observations only.<br>*The association observed between probable SWD and outcomes of health and work in this study is supported by several studies globally.* (p.24)<br><br>The conclusion has now been modified to address this concern.<br>*This study identified an unexpectedly high prevalence of probable SWD among healthcare shift workers, with 71% of surveyed shift workers meeting the criteria for probable SWD. Probable SWD was associated with poorer HRQoL, mental health, and work productivity. These findings highlight the substantial burden associated with probable SWD and support the need for accessible, targeted, and evidence-based interventions to address SWD among healthcare shift workers.* (p.26)

1.8	Consider briefly expanding on the generalisability of the findings beyond the NHS context.	The following sentence has been added at the start of the implications section to address this comment.<br>*Although the study was conducted in an NHS healthcare setting, the findings may be generalisable to other sectors that employ shift workers (e.g. transportation, emergency services, aviation), where similar work patterns and sleep challenges occur.* (p.25)
1.9	The manuscript provides general practical implications; however, these remain broad and could be further developed to enhance applicability.	Some specific actions have now been added in this section to address this comment.<br>*In practice, this may include integrating SWD screening into occupational health assessments, educating shift workers on recognising symptoms and healthy sleep behaviours, and training managers to support early identification and referral. Clear referral pathways will also be needed to facilitate timely diagnosis and treatment. Preventive strategies such as screening and managing sleep disorders before commencing shift work are now beginning to be considered to alleviate the impact of SWD.* (p.25)
**2**	**Reviewer 2**	
2.1	Please justify the definition of Working non-standard hours you used.	Thank you for providing the different definitions for non-standard working hours. We agree with you that there is no single agreed definition for non-standard working hours and therefore, we had to define this to ensure consistency across our programme of work on SWD. Non-standard working hours in this study refers to as work scheduled after 6 pm or before 7 am, and does not differentiate between week days and weekend. This includes night, early morning, late evening and rotating shifts. The specific timings were chosen on consideration of the varied shift schedules in the NHS.<br>A sentence has been added to the introduction to explain non-standard hours.<br>*This includes nights, early morning, late evening and rotating shifts.* (p.7)
2.2	Study design and sampling<br>How did authors select the prevalence rate of SWD of 20% for the calculation of the sample<br>size?	At the time of study planning, only the review by Pallesen et al. was available regarding estimates of SWD prevalence. This review reported an estimated SWD prevalence of 26.5% (95% CI: 21.0–32.8), with reported prevalence estimates

		ranging from 0% to 84%. Given this wide variation, a conservative prevalence estimate of 20% was used for the sample size calculation.<br>For clarity, the Pallesen et al. reference has now been added to the sample size calculation section. (p.8)
2.3	Statistical analyses<br>Please add the statistical tests performed in order the approximative sentence "using<br>appropriate statistical tests".	The sentence has been modified to include the specific tests.<br>*Differences between participants with and without probable SWD in demographic and survey outcome variables were evaluated using independent-samples t-tests or Mann–Whitney U tests for continuous variables, and chi-square tests or Fisher's exact tests for categorical variables.* (p.11)
2.4	In table 1, only the Fisher test is mentioned, but the comparison of Sleep duration (self-reported average per day over the last month) according to probably SWD must have been performed with a comparative quantitative test. Please add this information in the statistical part in methods.	As per the above response, the sentence has been modified to include the specific tests.<br>*Differences between participants with and without probable SWD in demographic and survey outcome variables were evaluated using independent-samples t-tests or Mann–Whitney U tests for continuous variables, and chi-square tests or Fisher's exact tests for categorical variables.* (p.11)<br>In Table 1, Fisher's exact test p-values were marked to distinguish them from p-values derived from other statistical tests.
2.5	The use of a cut-off at 2 and more for the Patient Health Questionnaire-2 in order to identify the likelihood of depression is not a good choice. Indeed, this cut-off maximizes sensitivity (detecting more potential cases), at the cost of a higher number of false positives. Authors have presented the cut-off at 3 and more. This cut-off must be used in the following of the manuscript and in the analyses.	Thank you for raising this. We would like to confirm that a cut-off of ≥3 was in fact used throughout the study for both the PHQ-2 and GAD-2. This is the cut-off recommended by the developers (Korenke 2003 and 2007). The appropriate references have also been used in the manuscript. We have checked through the manuscript to ensure there were no erroneous mentions of a cut off >2.<br>*The Patient Health Questionnaire-2 (PHQ-2) and the Generalized Anxiety Disorder-2 (GAD-2) were used to identify the likelihood of depression and anxiety, respectively. For both measures, total scores ranged from 0 to 6, with a score of ≥3 indicating a higher likelihood of current depression or anxiety.* (p.10)
2.6	Same comment for the estimation of the prevalence of anxiety, the cut-off 3 and more must be used in the analyses.	

2.7	For the UK EQ-5D-5L utility calculation, I thought that the correct reference was<br>Devlin NJ, Shah KK, Feng Y, Mulhern B, van Hout B. Valuing health-related quality of life: An EQ-5D-5L value set for England. *Health Economics*. 2018;27:7–22. https://doi.org/10.1002/hec.3564	The National Institute for Health and Care Excellence (NICE) in the UK does not recommend the use of Devlin et al. (2018) values. Instead, it recommends that utility values in reference-case analyses should be calculated by mapping the 5L descriptive system data onto the 3L value set and that the mapping function developed by the NICE Decision Support Unit, the University of Sheffield, (Hernández Alava et al. 2017), using the 'EEPRU dataset' (Hernández Alava et al. 2020), should be used for reference-case analyses (https://www.nice.org.uk/process/pmg36/chapter/economic-evaluation-2#evidence-on-resource-use-and-costs). We followed this recommended approach in the study and the relevant references were cited in the manuscript. The corresponding sentence in the manuscript has been modified for additional clarity.<br>*The responses to the EQ-5D-5L were converted into utility scores using the mapping function currently recommended by the UK NICE. (p.10)*<br><br>We are aware that a new UK EQ-5D-5L value set is now available, however, this value set was only released after the data analysis for this study had been completed and has not been recommended by NICE. Therefore, we used the recommended mapping approach in our study.
2.8	Results<br>It is not clear if the analyse of the Accidents/Near misses (motor vehicle, home or work related) due to sleepiness in the last 12 months has been performed on the sub population of participants who has Shift work duration more than 1 year. I don't think it is case regarding the number of total participants (n=443) in the table 1 for these 2 variables.	We would like to clarify that the analyses of accidents/near misses due to sleepiness were conducted in the full study sample and were not restricted to participants with a shift work duration of more than 12 months. Although the question referred to accidents/near misses occurring in the previous 12 months, all participants who provided a valid response were included in these analyses, regardless of their duration of shift work.
2.9	P16, ***Impact on eating habits (e.g., food choices and/or meal patterns)***<br>This paragraph is written like a discussion with an interpretation of the results but without references.	We would like to clarify that this section presents findings derived from participants' free-text responses and does not represent a discussion or interpretation of these findings. In line with your

	There are a lot of results presented, please keep concise paragraph for dimension studied.	feedback, we have revised this section to make it more concise.<br>*When asked to expand on their answers in free-text responses, participants in the probable SWD group reported that shift work and disrupted sleep led to irregular eating patterns. Fatigue, long shifts, missed or poorly timed breaks, and limited time to cook often led to reliance on convenience or processed foods. Participants also described fluctuating appetite, often linked to weight changes and digestive issues, while a small number reported using coping strategies to manage these challenges.* (p.18)
2.10	Discussion<br>The presence of probable SWD was associated with poorer HRQoL, work productivity, and<br>mental health. Why author add poorer for health quality of life, and stay general for work<br>productivity and mental health?	We would like to clarify that the term "poorer" was intended to apply to all three outcomes: HRQoL, work productivity, and mental health. We have now modified this sentence throughout the manuscript by adding 'outcomes' to the end of the sentence.<br>*Probable SWD was associated with poorer HRQoL, depression, anxiety, and work productivity outcomes.* (p.5, 23, 26)
2.11	Moreover, mental health is not relevant short resume of the depression and anxiety probable<br>issue. Indeed, mental health refers to a person's emotional, psychological, and social wellbeing,<br>not only depressive symptoms and anxiety symptoms. Some of these dimensions were not evaluated in this study.	We agree with the reviewer that mental health is a broad term and therefore, we have now replaced mental health with depression and anxiety.<br>*Probable SWD was associated with poorer HRQoL, depression, anxiety, and work productivity outcomes.* (p.5, 23, 26)
2.12	This study selected participants who worked shifts that involved working non-standard hours (defined as work scheduled after 6 pm or before 7 am and who worked non-standard hours<br>for a minimum of 3 months. Comparisons of prevalence of WSD with the 2 meta-analysis is quite difficult like these meta-analyses included several studies with several different definition of shift work. The criteria of selection if this study seems	We agree with the reviewer that this is not a like-for-like comparison. However, these two systematic reviews are the only recent reviews available that have summarised SWD prevalence estimates among shift workers and were therefore considered the most appropriate sources for comparison. We have acknowledged in the manuscript that there are important differences between studies, including the sectors in which shift workers were employed, the instruments used to estimate SWD, and the wide range of reported prevalence estimates (p.23-24).

	to be the more important and the more selective too. In this way, it is not surprising to find a higher prevalence of WSD. Moreover, the studies population in one of the meta-analyse included different type of workers, like this study focused on nurses.<br>The meta-analyse among nurses found a prevalence around 45%, with a lot of heterogeneity in shift works definition and also in the questionnaire used to evaluate the WSD.	We have also identified potential selection bias as a concern in both the discussion and limitations sections (p24, 26).
**3**	**Editor**	
3.1	Please revise the title of your manuscript to include the setting. This is the preferred format of the journal.	Heading modified to include the setting.<br>*Prevalence and associated burden of shift work disorder (SWD) in healthcare shift workers in the UK National Health Service (NHS)- A cross-sectional survey* (p.1)
3.2	Please amend the 'Outcome measure' heading to 'Primary and Secondary outcome measures' to align with our formatting guidelines http://bmjopen.bmj.com/pages/authors/#research	Done.
3.3	Please clarify if a priori sample size calculation was performed and please include this in the manuscript. If this was not performed, please explain why this is the case and please provide a rationale and/or justification for the sample size.	A priori sample size calculation was performed and this has been included in the manuscript under Study design and sampling.<br>*Based on an estimated prevalence rate of SWD of 20%, with a 95% confidence interval ranging from 16.3% to 24.1% calculated using the Clopper-Pearson method, a total sample size of 424 participants was determined to be required.* (p.8)
3.4	Please report the response rate if possible. By response rate, we are referring to the number of people who answered the survey divided by the number of people who were invited to take part in the survey. Please provide this data if known.	This information is already reported in the manuscript, as the first sentence of the Results section, which states:<br>*Due to the promotion and recruitment method used in the study, it was not possible to determine how many NHS staff had received information about the study, and thus the response denominator could not be established.* (p12)
3.5	Please delete the sections: What is already known on this topic, and What this study adds.	Done.

3.6	Articles should list each author's contribution individually at the end; this section may also include contributors who do not qualify as authors. Please visit the ICMJE website for more information on authorship.	Each author's contribution has now been added at the end of the manuscript. (p.28-29)

VERSION 2 - REVIEW

Reviewer **1**

Name **Dopelt, Keren**

Affiliation **Ben-Gurion University of the Negev, Health Policy and Management**

Date **05-Aug-2026**

COI

The authors have addressed the comments comprehensively and constructively.

Reviewer **2**

Name **Fort, Emmanuel**

Affiliation **University of Lyon, University of Lyon 1, University of Gustave Eiffel, UMRESTTE, UMR T_9405, F- 69373, LYON, France**

Date **07-Aug-2026**

COI

Dear Authors,

I thank you for having consider my comments and made the relevants modifications in the manuscrit.

I still have some few comments.

p10, authors wrote "The Patient Health Questionnaire-2 (PHQ-2)19 and the Generalized Anxiety Disorder-2 (GAD-2)20 were used to identify the likelihood of depression and anxiety, respectively. For both measures, total scores ranged from 0 to 6, with a score of ≥3 indicating a higher likelihood of current depression or anxiety."

I dont think that "higher likelihood" is well appropriated. Indeed, according to the meta analysis of Levis et al. (2020), the sensibility of the PHQ-2 for a cut-off at three was estimated to 0,72 (IC95 % : 0,67–0,77) and the specificity was estimated to 0,85 (IC95 % :

0,83–0,87). A sensitivity around 72% is not a higher sensibility but a correct one. Please add this limit in the discussion part and correct this sentence in the methods part.

In comparison, Levis and al. (2021) calculated a sensibility equal to 0,85 (and a specifity equal to 0,85) for a cut-off at 10 for the PHQ-9.

Using a short version of the PHQ is not a problem but this limit should be discuss.

Levis B, Sun Y, He C, et al. Accuracy of the PHQ-2 Alone and in Combination With the PHQ-9 for Screening to Detect Major Depression: Systematic Review and Meta-analysis. JAMA. 2020;323(22):2290–2300

Levis B, Benedetti A, Thombs BD, et al. Accuracy of the Patient Health Questionnaire-9 for screening to detect major depression: updated systematic review and individual participant data meta-analysis. BMJ. 2021;375:n2183

The same comment can be made for the limit of the GAD-2. The Cochrane review found a sensibity equal to 0,68 (IC95 % : 0,59–0,75).

Aktürk Z, Hapfelmeier A, Fomenko A, Dümmler D, Eck S, Olm M, Gehrmann J, von Schrottenberg V, Rehder R, Dawson S, Löwe B, Rücker G, Schneider A, Linde K. Generalized Anxiety Disorder 7-item (GAD-7) and 2-item (GAD-2) scales for detecting anxiety disorders in adults. Cochrane Database of Systematic Reviews 2025, Issue 3. Art. No.: CD015455. DOI: 10.1002/14651858.CD015455. Accessed 07 August 2026.

VERSION 2 - AUTHOR RESPONSE

	Reviewer comments	Author response
**1**	**Reviewer 2**	
1.10	p10, authors wrote "The Patient Health Questionnaire-2 (PHQ-2)19 and the Generalized Anxiety Disorder-2 (GAD-2)20 were used to identify the likelihood of depression and anxiety, respectively. For both measures, total scores ranged from 0 to 6, with a score of ≥3 indicating a higher likelihood of current depression or anxiety." I dont think that "higher likelihood" is well appropriated. Indeed, according to the meta analysis of Levis et al. (2020), the sensibility of the PHQ-2 for a cut-off at three was estimated to 0,72 (IC95 % : 0,67–0,77) and the specificity was estimated to 0,85 (IC95 % : 0,83–0,87). A sensitivity around	Thank you for this important comment and the references. We agree that the wording of the sentence in the methods section could be more precise and therefore the term 'higher likelihood' has been removed and the use of PHQ-2 and GAD-2 as screening instruments has been clarified.<br>*The Patient Health Questionnaire-2 (PHQ-2) and the Generalized Anxiety Disorder-2 (GAD-2) were used to screen for depressive and anxiety symptoms, respectively. For both measures, total scores ranged from 0 to 6, with a score of ≥3 indicating possible depression or anxiety.* (p.9)<br><br>As suggested, we have also acknowledged that the PHQ-2 and GAD-2 are screening tools and do not establish definitive diagnoses in the limitations section.

	72% is not a higher sensibility but a correct one. Please add this limit in the discussion part and correct this sentence in the methods part.<br><br>In comparison, Levis and al. (2021) calculated a sensibility equal to 0,85 (and a specifity equal to 0,85) for a cut-off at 10 for the PHQ-9.<br><br>Using a short version of the PHQ is not a problem but this limit should be discuss.<br><br>Levis B, Sun Y, He C, et al. Accuracy of the PHQ-2 Alone and in Combination With the PHQ-9 for Screening to Detect Major Depression: Systematic Review and Meta-analysis. JAMA. 2020;323(22):2290–2300<br>Levis B, Benedetti A, Thombs BD, et al. Accuracy of the Patient Health Questionnaire-9 for screening to detect major depression: updated systematic review and individual participant data meta-analysis. BMJ. 2021;375:n2183<br><br>The same comment can be made for the limit of the GAD-2. The Cochrane review found a sensibity equal to 0,68 (IC95 % : 0,59–0,75).<br><br>Aktürk Z, Hapfelmeier A, Fomenko A, Dümmler D, Eck S, Olm M, Gehrmann J, von Schrottenberg V, Rehder R, Dawson S, Löwe B, Rücker G, Schneider A, Linde K. Generalized Anxiety Disorder 7-item (GAD-7) and 2-item (GAD-2) scales for detecting anxiety disorders in adults. Cochrane Database of Systematic Reviews 2025, Issue 3. Art. No.: CD015455. DOI: 10.1002/14651858.CD015455. Accessed 07 August 2026.	*Similarly, the PHQ-2 and GAD-2 are screening tools and do not establish definitive diagnoses of depression and anxiety, respectively.* (p.24)

VERSION 3 - REVIEW

**Reviewer**	**1**
**Name**	**Dopelt, Keren**
**Affiliation**	**Ben-Gurion University of the Negev, Health Policy and Management**
**Date**	**13-Aug-2026**
**COI**	

The authors have addressed all the comments.

**Reviewer**	**2**
**Name**	**Fort, Emmanuel**
**Affiliation**	**University of Lyon, University of Lyon 1, University of Gustave Eiffel, UMRESTTE, UMR T_9405, F- 69373, LYON, France**
**Date**	**13-Aug-2026**
**COI**	

Dear authors,

thank you for this last minor corrections.
