# Supplementary material for: Prevalence and associated burden of shift work disorder (SWD) in healthcare shift workers in the UK National Health Service (NHS): a cross-sectional survey
Source: BMJ Open. 2026 Nov 5;16(11):e118265. doi: 10.1136/bmjopen-2026-118265 (PMC13756277; doi:10.1136/bmjopen-2026-118265)
Supplement: Supplementary file 2 [file bmjopen-16-11-s001.docx]

**Supplementary Materials**

# Supplementary material 1: Sensitivity analysis for EQ-5D-5L utility scores - Methods

First, an ordinary least squares (OLS) regression model with bootstrap standard errors was estimated to obtain inference robust to deviations from normality and heteroscedasticity. Second, a beta regression model with a logit link was fitted to account for the bounded and skewed distribution of utility values. The beta regression excluded one participant with a negative utility value, as this observation lay outside the theoretical support of the beta distribution (0,1). In addition, a precision sub-model was specified because residual diagnostics from a beta regression without a precision component indicated potential systematic heteroscedasticity in the utility scores.

# Supplementary material 2: Strengthening the Reporting of Observational Studies in Epidemiology (STROBE) statement

**S Table 1: STROBE Statement—Checklist of items that should be included in reports of *cross-sectional studies***

|  | **Item No** | **Recommendation** | **Page No** |
| --- | --- | --- | --- |
| **Title and abstract** | 1 | (*a*) Indicate the study’s design with a commonly used term in the title or the abstract | 1, 3 |
|  |  | (*b*) Provide in the abstract an informative and balanced summary of what was done and what was found | 3-4 |
| **Introduction** | | | |
| Background/rationale | 2 | Explain the scientific background and rationale for the investigation being reported | 6 |
| Objectives | 3 | State specific objectives, including any prespecified hypotheses | 6-7 |
| **Methods** | | | |
| Study design | 4 | Present key elements of study design early in the paper | 7 |
| Setting | 5 | Describe the setting, locations, and relevant dates, including periods of recruitment, exposure, follow-up, and data collection | 7 |
| Participants | 6 | (*a*) Give the eligibility criteria, and the sources and methods of selection of participants | 7-8 |
| Variables | 7 | Clearly define all outcomes, exposures, predictors, potential confounders, and effect modifiers. Give diagnostic criteria, if applicable | 8-9 |
| Data sources/ measurement | 8 | For each variable of interest, give sources of data and details of methods of assessment (measurement). Describe comparability of assessment methods if there is more than one group | 8-9 |
| Bias | 9 | Describe any efforts to address potential sources of bias | 10 |
| Study size | 10 | Explain how the study size was arrived at | 7 |
| Quantitative variables | 11 | Explain how quantitative variables were handled in the analyses. If applicable, describe which groupings were chosen and why | 9-10 |
| Statistical methods | 12 | (*a*) Describe all statistical methods, including those used to control for confounding | 9-10 & Supplementary material 1 |
|  |  | (*b*) Describe any methods used to examine subgroups and interactions | 9-10 |
|  |  | (*c*) Explain how missing data were addressed | 9-10 |
|  |  | (*d*) If applicable, describe analytical methods taking account of sampling strategy | NA |
|  |  | (*e*) Describe any sensitivity analyses | 9-10 & Supplementary material 1 |
| **Results** | | | |
| Participants | 13 | (a) Report numbers of individuals at each stage of study—eg numbers potentially eligible, examined for eligibility, confirmed eligible, included in the study, completing follow-up, and analysed | 11 |
|  |  | (b) Give reasons for non-participation at each stage | 11 |
|  |  | (c) Consider use of a flow diagram | 11 |
| Descriptive data | 14 | (a) Give characteristics of study participants (eg demographic, clinical, social) and information on exposures and potential confounders | 11-12 |
|  |  | (b) Indicate number of participants with missing data for each variable of interest | 13-14 &17 |
| Outcome data | 15 | Report numbers of outcome events or summary measures | 12-16 |
| Main results | 16 | (*a*) Give unadjusted estimates and, if applicable, confounder-adjusted estimates and their precision (eg, 95% confidence interval). Make clear which confounders were adjusted for and why they were included | 12-16 |
|  |  | (*b*) Report category boundaries when continuous variables were categorized | 13-14 &17 |
|  |  | (*c*) If relevant, consider translating estimates of relative risk into absolute risk for a meaningful time period | NA |
| Other analyses | 17 | Report other analyses done—eg analyses of subgroups and interactions, and sensitivity analyses | 17-21 & Supplementary material 4 |
| **Discussion** | | | |
| Key results | 18 | Summarise key results with reference to study objectives | 21 |
| Limitations | 19 | Discuss limitations of the study, taking into account sources of potential bias or imprecision. Discuss both direction and magnitude of any potential bias | 24 |
| Interpretation | 20 | Give a cautious overall interpretation of results considering objectives, limitations, multiplicity of analyses, results from similar studies, and other relevant evidence | 21-24 |
| Generalisability | 21 | Discuss the generalisability (external validity) of the study results | 21-23 |
| **Other information** | | | |
| Funding | 22 | Give the source of funding and the role of the funders for the present study and, if applicable, for the original study on which the present article is based | 26 |

# Supplementary material 3: Characteristics of participants differentiated by sites

S Table 2: Characteristics of participants differentiated by sites

| **Variables** | | **All participants** | **Site 1** | **Site 2** | **p value** |
| --- | --- | --- | --- | --- | --- |
| Number of participants (%) | | 443 | 248 (54%) | 195 (43%) |  |
| Age in years (n=443)  Mean ± SD | | 36.09±10.71 | 36.39±10.67 | 35.72±10.77 | 0.51 |
| Sex (n=443) | Female | 366 (83%) | 199 (80%) | 167 (86%) | 0.12 |
|  | Male | 74 (17%) | 48 (19%) | 26 (13%) |  |
|  | Non binary† | 1 | 0 | 1 |  |
|  | Prefer not to say† | 2 | 1 | 1 |  |
| Employment type (n=443) | Full-time (37.5 or more hours/week) | 320 (72%) | 215 (87%) | 105 (54%) | **<0.001*** |
|  | Part-time (less than 37.5 hours/week) | 123 (28%) | 33 (13%) | 90 (46%) |  |
| NHS job band (n=443) | Band 2 | 25 (6%) | 2 (1%) | 23 (12%) | **<0.001*** |
|  | Band 3 | 61 (14%) | 30 (12%) | 31 (16%) |  |
|  | Band 4 | 27 (6%) | 14 (6%) | 13 (7%) |  |
|  | Band 5 | 120 (27%) | 62 (25%) | 58 (30%) |  |
|  | Band 6 | 135 (31%) | 90 (36%) | 45 (23%) |  |
|  | Band 7 and above | 35 (8%) | 27 (11%) | 8 (4%) |  |
|  | Other | 36 (8%) | 22 (9%) | 14 (7%) |  |
|  | Prefer not to say† | 4 (1%) | 1 | 3 (2%) |  |
| Occupational role‡ (n=443) | Medical / Dental | 39 (9%) | 23 (9%) | 16 (8%) | **<0.001*** |
|  | Registered Nurse | 218 (49%) | 131 (53%) | 87(45%) |  |
|  | Midwives | 43 (10%) | 32 (13%) | 11 (6%) |  |
|  | Nursing auxiliary / Nursing assistant / Healthcare assistant | 45 (10%) | 11 (4%) | 34 (17%) |  |
|  | Admin and clerical | 50 (11%) | 30 (12%) | 20 (10%) |  |
|  | Wider health care team (e.g. Maintenance, Ambulance) | 9 (2%) | 3 (1%) | 6 (3%) |  |
|  | Allied health professionals / Healthcare Scientists / Scientific and Technical | 39 (9%) | 18 (7%) | 21 (11%) |  |
| Ethnicity‡ (n=443) | White: English/Welsh/Scottish/Northern Irish/British/Irish/Other | 196 (46%) | 95 (38%) | 101 (52%) | **<0.001*** |
|  | Asian/Asian British | 143 (33%) | 79 (32%) | 64 (33%) |  |
|  | Black / African / Caribbean / Black British | 54 (13%) | 38 (15%) | 16 (8%) |  |
|  | Mixed/Multiple ethnic group | 19 (4%) | 11 (4%) | 8 (4%) |  |
|  | Other ethnic groups | 18 (4%) | 17 (7%) | 1 (1%) |  |
|  | Prefer not to say† | 13 (3%) | 8 (3%) | 5 (3%) |  |
| Type of shift work (n=443) | Evening/late shifts (work usually finished between 6pm and midnight) | 63 (14%) | 44 (18%) | 19 (10%) | **<0.001*^§^** |
|  | Morning shifts (work usually started before 7am) | 6 (1%) | 2 (1%) | 4 (2%) |  |
|  | Night shifts (work hours included at least 3 hours between 11pm and 6am) | 88 (20%) | 35 (14%) | 53 (27%) |  |
|  | Rotating shifts (periodically changed or had irregular work hours) | 286 (65%) | 167 (67%) | 119 (61%) |  |
| Shift work duration (n=443) | Less than 1 year | 35 (8%) | 18 (7%) | 17 (8%) | 0.13 |
|  | 1–5 years | 196 (44%) | 99 (40%) | 97 (50%) |  |
|  | 6–10 years | 101 (23%) | 67 (27%) | 34 (17%) |  |
|  | 11–20 years | 78 (18%) | 45 (18%) | 33 (17%) |  |
|  | More than 20 years | 33 (7%) | 19 (8%) | 14 (7%) |  |
| Self-report of sleep problems and duration (n=443) | I don’t have a problem | 80 (18%) | 41 (17%) | 39 (20%) | 0.91 |
|  | Less than one month | 22 (5%) | 12 (5%) | 10 (5%) |  |
|  | 1-2 months | 24 (5%) | 14 (6%) | 10 (5%) |  |
|  | 3-6 months | 46 (10%) | 24 (10%) | 22 (11%) |  |
|  | 7-12 months | 30 (7%) | 17 (7%) | 13 (7%) |  |
|  | More than 1 year | 241 (54%) | 140 (57%) | 101 (52%) |  |
| Accidents/Near misses (motor vehicle, home or work related) due to sleepiness (n=443) | 0 accidents | 244 (55%) | 140 (47%) | 104 (53%) | 0.61^§^ |
|  | 1–5 accidents | 121 (27%) | 68 (27%) | 53 (27%) |  |
|  | 6–10 accidents | 7 (2%) | 5 (2%) | 2 (1%) |  |
|  | More than 10 accidents | 7 (2%) | 5 (2%) | 2 (1%) |  |
|  | Do not know | 53 (12%) | 25 (10%) | 28 (14%) |  |
|  | Prefer not to answer | 11 (3%) | 5 (2%) | 6 (3%) |  |
| Sleep duration (self-reported average per day over the last month) (n=432)  Mean ± SD | | 6.2±1.2 | 6.3±1.2 | 6.2±1.2 | 0. 64 |
| Presence of long-term health condition (n=443) | Yes | 165 (37%) | 92 (37%) | 73 (37%) | 0.93 |
|  | No | 258 (58%) | 145 (59%) | 113 (58%) |  |
|  | Prefer not to answer† | 20 (5%) | 11 (4%) | 9 (5%) |  |

Key: *- Statistically significant difference; †- Not included in the statistical analysis; ‡- Categories have been collapsed to broader top-level groups for ease of reporting; All percentages presented are based on the within group value for that particular variable; §- Fisher's exact p.

# Supplementary material 4: Results of regression analyses

S Table 3: EQ-5D-5L utility scores (outcome variable) and probable SWD status (main explanatory variable) Model 1

Adjusted R^2^=0.29, p<0.001, n=400

| **Variables** | **Unstandardized coefficient** | | **Standardized Coefficients β** | **t** | **95% CI** | | **p** |
| --- | --- | --- | --- | --- | --- | --- | --- |
|  | **B** | **Standard error** |  |  | **Lower** | **Upper** |  |
| Probable SWD status | -0.08 | 0.02 | -0.18 | -4.12 | -0.12 | -0.04 | **<0.001*** |
| Site |  |  |  |  |  |  |  |
| Site 1 | Ref | - | - | - | - | - | - |
| Site 2 | 0.05 | 0.02 | 0.13 | 2.85 | 0.02 | 0.09 | **0.01*** |
| Age | 0.00 | 0.00 | -0.07 | -1.64 | 0.00 | 0.00 | 0.10 |
| Gender |  |  |  |  |  |  |  |
| Male | Ref | - | - | - | - | - | - |
| Female | -0.05 | 0.02 | -0.09 | -2.05 | -0.10 | 0.00 | **0.04*** |
| Ethnicity |  |  |  |  |  |  |  |
| White | Ref | - | - | - | - | - | - |
| Asian/Asian British | 0.01 | 0.02 | 0.03 | 0.66 | -0.03 | 0.05 | 0.51 |
| Black / Black British | -0.01 | 0.03 | -0.02 | -0.46 | -0.06 | 0.04 | 0.64 |
| Mixed/Multiple ethnic group | -0.03 | 0.04 | -0.03 | -0.67 | -0.11 | 0.05 | 0.51 |
| Other ethnic groups | 0.00 | 0.05 | 0.00 | -0.09 | -0.10 | 0.09 | 0.93 |
| Occupational role |  |  |  |  |  |  |  |
| Medical / Dental | Ref | - | - | - | - | - | - |
| Registered Nurse | -0.02 | 0.03 | -0.05 | -0.60 | -0.08 | 0.04 | 0.55 |
| Midwives | 0.02 | 0.04 | 0.04 | 0.59 | -0.05 | 0.10 | 0.56 |
| Nursing auxiliary / Nursing assistant / Healthcare assistant | -0.08 | 0.04 | -0.11 | -1.88 | -0.16 | 0.00 | 0.06 |
| Admin and clerical | -0.11 | 0.04 | -0.19 | -2.98 | -0.19 | -0.04 | **0.00*** |
| Wider health care team (e.g. Maintenance, Ambulance) | -0.13 | 0.07 | -0.10 | -1.98 | -0.26 | 0.00 | **0.05*** |
| Allied health professionals / Healthcare Scientists / Scientific and Technical | -0.04 | 0.04 | -0.05 | -0.88 | -0.12 | 0.04 | 0.38 |
| Presence of long-term health condition |  |  |  |  |  |  |  |
| No | Ref | - | - | - | - | - | - |
| Yes | -0.14 | 0.02 | -0.35 | -7.77 | -0.17 | -0.10 | **<0.001*** |
| Self-reported sleep duration | 0.03 | 0.01 | 0.20 | 4.50 | 0.02 | 0.05 | **<0.001*** |
| (Constant) | 0.79 | 0.07 |  | 11.00 | 0.65 | 0.93 | **<0.001*** |

Key: *- Statistically significant difference

S Table 4: EQ-5D-5L utility scores (outcome variable) and probable SWD status (main explanatory variable) Model 2

Adjusted R^2^=0.29, p<0.001, n=400

| **Variables** | **Unstandardized coefficient** | | **Standardized Coefficients β** | **t** | **95% CI** | | **p** |
| --- | --- | --- | --- | --- | --- | --- | --- |
|  | **B** | **Standard error** |  |  | **Lower** | **Upper** |  |
| Probable SWD status | -0.08 | 0.02 | -0.18 | -4.02 | -0.12 | -0.04 | **<0.001*** |
| Site |  |  |  |  |  |  |  |
| Site 1 | Ref | - | - | - | - | - | - |
| Site 2 | 0.05 | 0.02 | 0.12 | 2.55 | 0.01 | 0.09 | **0.01*** |
| Age | 0.00 | 0.00 | -0.13 | -2.20 | 0.00 | 0.00 | **0.03*** |
| Gender |  |  |  |  |  |  |  |
| Male | Ref | - | - | - | - | - | - |
| Female | -0.05 | 0.02 | -0.10 | -2.15 | -0.10 | 0.00 | **0.03*** |
| Ethnicity |  |  |  |  |  |  |  |
| White | Ref | - | - | - | - | - | - |
| Asian/Asian British | 0.02 | 0.02 | 0.04 | 0.79 | -0.02 | 0.06 | 0.43 |
| Black / Black British | 0.00 | 0.03 | -0.01 | -0.14 | -0.06 | 0.05 | 0.89 |
| Mixed/Multiple ethnic group | -0.02 | 0.04 | -0.02 | -0.53 | -0.10 | 0.06 | 0.59 |
| Other ethnic groups | 0.01 | 0.05 | 0.01 | 0.18 | -0.08 | 0.10 | 0.86 |
| Occupational role |  |  |  |  |  |  |  |
| Medical / Dental | Ref | - | - | - | - | - | - |
| Registered Nurse | -0.02 | 0.03 | -0.06 | -0.78 | -0.08 | 0.04 | 0.44 |
| Midwives | 0.01 | 0.04 | 0.02 | 0.36 | -0.07 | 0.09 | 0.72 |
| Nursing auxiliary / Nursing assistant / Healthcare assistant | -0.08 | 0.04 | -0.12 | -1.93 | -0.16 | 0.00 | **0.05*** |
| Admin and clerical | -0.11 | 0.04 | -0.18 | -2.73 | -0.18 | -0.03 | **0.01*** |
| Wider health care team (e.g. Maintenance, Ambulance) | -0.13 | 0.07 | -0.10 | -1.97 | -0.27 | 0.00 | **0.05*** |
| Allied health professionals / Healthcare Scientists / Scientific and Technical | -0.03 | 0.04 | -0.05 | -0.82 | -0.11 | 0.05 | 0.41 |
| Presence of long-term health condition |  |  |  |  |  |  |  |
| No | Ref | - | - | - | - | - | - |
| Yes | -0.14 | 0.02 | -0.34 | -7.54 | -0.17 | -0.10 | **<0.001*** |
| Self-reported sleep duration | 0.03 | 0.01 | 0.20 | 4.49 | 0.02 | 0.05 | **<0.001*** |
| Employment type |  |  |  |  |  |  |  |
| Full-time | Ref | - | - | - | - | - | - |
| Part-time | 0.00 | 0.02 | 0.01 | 0.17 | -0.04 | 0.05 | 0.86 |
| Duration of working shifts (years) | 0.00 | 0.00 | 0.06 | 1.09 | 0.00 | 0.00 | 0.28 |
| Shift type |  |  |  |  |  |  |  |
| Rotating shifts | Ref | - | - | - | - | - | - |
| Evening shift | 0.03 | 0.03 | 0.06 | 1.24 | -0.02 | 0.08 | 0.22 |
| Night shift | 0.04 | 0.02 | 0.07 | 1.55 | -0.01 | 0.08 | 0.12 |
| (Constant) | 0.80 | 0.07 |  | 10.81 | 0.66 | 0.95 | **<0.001*** |

Key: *- Statistically significant difference

S Table 5: EQ-5D-5L VAS scores (outcome variable) and probable SWD status (main explanatory variable) Model 1

Adjusted R^2^=0.18, p<0.001, n=400

| **Variables** | **Unstandardized coefficient** | | **Standardized Coefficients β** | **t** | **95% CI** | | **p** |
| --- | --- | --- | --- | --- | --- | --- | --- |
|  | **B** | **Standard error** |  |  | **Lower** | **Upper** |  |
| Probable SWD status | -5.75 | 2.09 | -0.13 | -2.75 | -9.86 | -1.64 | **0.01*** |
| Site |  |  |  |  |  |  |  |
| Site 1 | Ref | - | - | - | - | - | - |
| Site 2 | -0.48 | 1.91 | -0.01 | -0.25 | -4.25 | 3.28 | 0.80 |
| Age | 0.06 | 0.09 | 0.03 | 0.67 | -0.11 | 0.23 | 0.50 |
| Gender |  |  |  |  |  |  |  |
| Male | Ref | - | - | - | - | - | - |
| Female | -1.86 | 2.60 | -0.04 | -0.72 | -6.98 | 3.25 | 0.48 |
| Ethnicity |  |  |  |  |  |  |  |
| White | Ref | - | - | - | - | - | - |
| Asian/Asian British | 3.14 | 2.20 | 0.07 | 1.43 | -1.19 | 7.47 | 0.15 |
| Black / Black British | 4.05 | 2.84 | 0.07 | 1.43 | -1.54 | 9.63 | 0.16 |
| Mixed/Multiple ethnic group | 3.72 | 4.32 | 0.04 | 0.86 | -4.77 | 12.20 | 0.39 |
| Other ethnic groups | 4.48 | 5.02 | 0.04 | 0.89 | -5.40 | 14.36 | 0.37 |
| Occupational role |  |  |  |  |  |  |  |
| Medical / Dental | Ref | - | - | - | - | - | - |
| Registered Nurse | -1.83 | 3.29 | -0.05 | -0.55 | -8.30 | 4.65 | 0.58 |
| Midwives | 3.07 | 4.23 | 0.05 | 0.72 | -5.26 | 11.39 | 0.47 |
| Nursing auxiliary / Nursing assistant / Healthcare assistant | -3.93 | 4.36 | -0.06 | -0.90 | -12.50 | 4.65 | 0.37 |
| Admin and clerical | -8.78 | 4.08 | -0.14 | -2.15 | -16.79 | -0.76 | **0.03*** |
| Wider health care team (e.g. Maintenance, Ambulance) | -19.67 | 7.20 | -0.14 | -2.73 | -33.82 | -5.51 | **0.01*** |
| Allied health professionals / Healthcare Scientists / Scientific and Technical | -5.68 | 4.38 | -0.08 | -1.30 | -14.28 | 2.93 | 0.20 |
| Presence of long-term health condition |  |  |  |  |  |  |  |
| No | Ref | - | - | - | - | - | - |
| Yes | -10.36 | 1.92 | -0.26 | -5.40 | -14.13 | -6.58 | **<0.001*** |
| Self-reported sleep duration | 3.79 | 0.79 | 0.23 | 4.80 | 2.24 | 5.35 | **<0.001*** |
| (Constant) | 56.88 | 7.79 |  | 7.31 | 41.57 | 72.19 | **<0.001*** |

Key: *- Statistically significant difference

S Table 6: EQ-5D-5L VAS scores (outcome variable) and probable SWD status (main explanatory variable) Model 2

Adjusted R^2^=0.18, p<0.001, n=400

| **Variables** | **Unstandardized coefficient** | | **Standardized Coefficients β** | **t** | **95% CI** | | **p** |
| --- | --- | --- | --- | --- | --- | --- | --- |
|  | **B** | **Standard error** |  |  | **Lower** | **Upper** |  |
| Probable SWD status | -5.76 | 2.13 | -0.13 | -2.70 | -9.95 | -1.57 | **0.01*** |
| Site |  |  |  |  |  |  |  |
| Site 1 | Ref | - | - | - | - | - | - |
| Site 2 | -0.70 | 2.07 | -0.02 | -0.34 | -4.77 | 3.37 | 0.74 |
| Age | 0.01 | 0.11 | 0.00 | 0.05 | -0.21 | 0.22 | 0.96 |
| Gender |  |  |  |  |  |  |  |
| Male | Ref | - | - | - | - | - | - |
| Female | -1.98 | 2.62 | -0.04 | -0.76 | -7.12 | 3.16 | 0.45 |
| Ethnicity |  |  |  |  |  |  |  |
| White | Ref | - | - | - | - | - | - |
| Asian/Asian British | 3.37 | 2.26 | 0.08 | 1.49 | -1.07 | 7.80 | 0.14 |
| Black / Black British | 4.59 | 2.93 | 0.08 | 1.57 | -1.17 | 10.34 | 0.12 |
| Mixed/Multiple ethnic group | 4.01 | 4.35 | 0.04 | 0.92 | -4.55 | 12.57 | 0.36 |
| Other ethnic groups | 5.16 | 5.09 | 0.05 | 1.01 | -4.84 | 15.15 | 0.31 |
| Occupational role |  |  |  |  |  |  |  |
| Medical / Dental | Ref | - | - | - | - | - | - |
| Registered Nurse | -2.07 | 3.33 | -0.05 | -0.62 | -8.63 | 4.48 | 0.53 |
| Midwives | 2.54 | 4.36 | 0.04 | 0.58 | -6.04 | 11.11 | 0.56 |
| Nursing auxiliary / Nursing assistant / Healthcare assistant | -4.13 | 4.45 | -0.06 | -0.93 | -12.88 | 4.63 | 0.36 |
| Admin and clerical | -8.30 | 4.20 | -0.14 | -1.98 | -16.55 | -0.05 | **0.05*** |
| Wider health care team (e.g. Maintenance, Ambulance) | -19.45 | 7.32 | -0.14 | -2.66 | -33.84 | -5.06 | **0.01*** |
| Allied health professionals / Healthcare Scientists / Scientific and Technical | -5.45 | 4.45 | -0.08 | -1.22 | -14.20 | 3.31 | 0.22 |
| Presence of long-term health condition |  |  |  |  |  |  |  |
| No | Ref | - | - | - | - | - | - |
| Yes | -10.23 | 1.95 | -0.26 | -5.25 | -14.06 | -6.40 | **<0.001*** |
| Self-reported sleep duration | 3.80 | 0.80 | 0.23 | 4.75 | 2.23 | 5.38 | **<0.001*** |
| Employment type |  |  |  |  |  |  |  |
| Full-time | Ref | - | - | - | - | - | - |
| Part-time | 0.51 | 2.43 | 0.01 | 0.21 | -4.26 | 5.29 | 0.83 |
| Duration of working shifts (years) | 0.10 | 0.16 | 0.04 | 0.60 | -0.22 | 0.41 | 0.55 |
| Shift type |  |  |  |  |  |  |  |
| Rotating shifts | Ref | - | - | - | - | - | - |
| Evening shift | 1.16 | 2.72 | 0.02 | 0.43 | -4.18 | 6.50 | 0.67 |
| Night shift | 1.67 | 2.44 | 0.04 | 0.69 | -3.12 | 6.46 | 0.49 |
| (Constant) | 57.42 | 8.05 |  | 7.13 | 41.59 | 73.25 | **<0.001*** |

Key: *- Statistically significant difference

S Table 7: Presenteeism scores (outcome variable) and probable SWD status (main explanatory variable) Model 1

Adjusted R^2^=0.07, p<0.001, n=312

| **Variables** | **Unstandardized coefficient** | | **Standardized Coefficients β** | **t** | **95% CI** | | **p** |
| --- | --- | --- | --- | --- | --- | --- | --- |
|  | **B** | **Standard error** |  |  | **Lower** | **Upper** |  |
| Probable SWD status | 9.72 | 3.07 | 0.18 | 3.17 | 3.69 | 15.75 | **0.002*** |
| Site |  |  |  |  |  |  |  |
| Site 1 | Ref | - | - | - | - | - | - |
| Site 2 | -3.93 | 2.74 | -0.09 | -1.44 | -9.31 | 1.46 | 0.15 |
| Age | -0.11 | 0.13 | -0.05 | -0.83 | -0.36 | 0.15 | 0.41 |
| Gender |  |  |  |  |  |  |  |
| Male | Ref | - | - | - | - | - | - |
| Female | 3.57 | 3.76 | 0.06 | 0.95 | -3.82 | 10.96 | 0.34 |
| Ethnicity |  |  |  |  |  |  |  |
| White | Ref | - | - | - | - | - | - |
| Asian/Asian British | 0.83 | 3.16 | 0.02 | 0.26 | -5.38 | 7.03 | 0.79 |
| Black / Black British | -3.45 | 4.23 | -0.05 | -0.81 | -11.78 | 4.89 | 0.42 |
| Mixed/Multiple ethnic group | 1.43 | 6.03 | 0.01 | 0.24 | -10.44 | 13.30 | 0.81 |
| Other ethnic groups | 19.05 | 6.77 | 0.17 | 2.81 | 5.73 | 32.37 | **0.01*** |
| Occupational role |  |  |  |  |  |  |  |
| Medical / Dental | Ref | - | - | - | - | - | - |
| Registered Nurse | -11.87 | 4.43 | -0.26 | -2.68 | -20.59 | -3.16 | **0.01*** |
| Midwives | -7.99 | 5.64 | -0.11 | -1.42 | -19.08 | 3.11 | 0.16 |
| Nursing auxiliary / Nursing assistant / Healthcare assistant | -10.06 | 6.06 | -0.13 | -1.66 | -21.98 | 1.86 | 0.10 |
| Admin and clerical | -10.83 | 5.96 | -0.14 | -1.82 | -22.56 | 0.90 | 0.07 |
| Wider health care team (e.g. Maintenance, Ambulance) | -12.73 | 9.70 | -0.08 | -1.31 | -31.82 | 6.36 | 0.19 |
| Allied health professionals / Healthcare Scientists / Scientific and Technical | -5.21 | 5.86 | -0.07 | -0.89 | -16.74 | 6.32 | 0.37 |
| Presence of long-term health condition |  |  |  |  |  |  |  |
| No | Ref | - | - | - | - | - | - |
| Yes | 4.23 | 2.73 | 0.09 | 1.55 | -1.14 | 9.60 | 0.12 |
| Self-reported sleep duration | -0.60 | 1.15 | -0.03 | -0.52 | -2.87 | 1.67 | 0.61 |
| (Constant) | 43.39 | 11.27 |  | 3.85 | 21.21 | 65.57 | **<0.001*** |

Key: *- Statistically significant difference

S Table 8: Presenteeism (outcome variable) and probable SWD status (main explanatory variable) Model 2

Adjusted R^2^=0.07, p=0.002, n=312

| **Variables** | **Unstandardized coefficient** | | **Standardized Coefficients β** | **t** | **95% CI** | | **p** |
| --- | --- | --- | --- | --- | --- | --- | --- |
|  | **B** | **Standard error** |  |  | **Lower** | **Upper** |  |
| Probable SWD status | 9.43 | 3.11 | 0.18 | 3.03 | 3.31 | 15.54 | **0.003*** |
| Site |  |  |  |  |  |  |  |
| Site 1 | Ref | - | - | - | - | - | - |
| Site 2 | -2.12 | 2.92 | -0.05 | -0.72 | -7.87 | 3.64 | 0.47 |
| Age | -0.09 | 0.17 | -0.04 | -0.53 | -0.42 | 0.24 | 0.59 |
| Gender |  |  |  |  |  |  |  |
| Male | Ref | - | - | - | - | - | - |
| Female | 3.86 | 3.77 | 0.06 | 1.03 | -3.55 | 11.28 | 0.31 |
| Ethnicity |  |  |  |  |  |  |  |
| White | Ref | - | - | - | - | - | - |
| Asian/Asian British | 0.51 | 3.24 | 0.01 | 0.16 | -5.87 | 6.88 | 0.88 |
| Black / Black British | -4.79 | 4.37 | -0.07 | -1.10 | -13.39 | 3.81 | 0.27 |
| Mixed/Multiple ethnic group | 0.34 | 6.06 | 0.00 | 0.06 | -11.58 | 12.26 | 0.96 |
| Other ethnic groups | 17.37 | 6.83 | 0.15 | 2.54 | 3.93 | 30.81 | 0.01 |
| Occupational role |  |  |  |  |  |  |  |
| Medical / Dental | Ref | - | - | - | - | - | - |
| Registered Nurse | -11.98 | 4.47 | -0.26 | -2.68 | -20.78 | -3.18 | **0.01*** |
| Midwives | -5.96 | 5.76 | -0.08 | -1.04 | -17.30 | 5.37 | 0.30 |
| Nursing auxiliary / Nursing assistant / Healthcare assistant | -8.53 | 6.13 | -0.11 | -1.39 | -20.60 | 3.54 | 0.17 |
| Admin and clerical | -11.92 | 6.13 | -0.15 | -1.95 | -23.98 | 0.14 | **0.05*** |
| Wider health care team (e.g. Maintenance, Ambulance) | -13.85 | 9.85 | -0.09 | -1.41 | -33.24 | 5.54 | 0.16 |
| Allied health professionals / Healthcare Scientists / Scientific and Technical | -6.90 | 5.96 | -0.09 | -1.16 | -18.62 | 4.83 | 0.25 |
| Presence of long-term health condition |  |  |  |  |  |  |  |
| No | Ref | - | - | - | - | - | - |
| Yes | 4.03 | 2.76 | 0.09 | 1.46 | -1.39 | 9.45 | 0.15 |
| Self-reported sleep duration | -0.80 | 1.17 | -0.04 | -0.68 | -3.10 | 1.50 | 0.50 |
| Employment type |  |  |  |  |  |  |  |
| Full-time | Ref | - | - | - | - | - | - |
| Part-time | -4.76 | 3.36 | -0.10 | -1.42 | -11.37 | 1.85 | 0.16 |
| Duration of working shifts (years) | 0.04 | 0.23 | 0.01 | 0.15 | -0.42 | 0.49 | 0.88 |
| Shift type |  |  |  |  |  |  |  |
| Rotating shifts | Ref | - | - | - | - | - | - |
| Evening shift | 1.44 | 3.92 | 0.02 | 0.37 | -6.27 | 9.15 | 0.71 |
| Night shift | -4.10 | 3.48 | -0.07 | -1.18 | -10.95 | 2.76 | 0.24 |
| (Constant) | 45.35 | 11.77 |  | 3.85 | 22.17 | 68.52 | **<0.001*** |

Key: *- Statistically significant difference

S Table 9: Overall work impairment (outcome variable) and probable SWD status (main explanatory variable) Model 1

Adjusted R^2^=0.06, p=0.003, n=311

| **Variables** | **Unstandardized coefficient** | | **Standardized Coefficients β** | **t** | **95% CI** | | **p** |
| --- | --- | --- | --- | --- | --- | --- | --- |
|  | **B** | **Standard error** |  |  | **Lower** | **Upper** |  |
| Probable SWD status | 9.91 | 3.26 | 0.18 | 3.05 | 3.50 | 16.32 | **0.003*** |
| Site |  |  |  |  |  |  |  |
| Site 1 | Ref | - | - | - | - | - | - |
| Site 2 | -4.92 | 2.91 | -0.10 | -1.69 | -10.65 | 0.80 | 0.09 |
| Age | -0.12 | 0.14 | -0.05 | -0.87 | -0.39 | 0.15 | 0.39 |
| Gender |  |  |  |  |  |  |  |
| Male | Ref | - | - | - | - | - | - |
| Female | 3.51 | 3.98 | 0.05 | 0.88 | -4.32 | 11.34 | 0.38 |
| Ethnicity |  |  |  |  |  |  |  |
| White | Ref | - | - | - | - | - | - |
| Asian/Asian British | 1.42 | 3.34 | 0.03 | 0.42 | -5.16 | 7.99 | 0.67 |
| Black / Black British | -1.80 | 4.49 | -0.02 | -0.40 | -10.63 | 7.02 | 0.69 |
| Mixed/Multiple ethnic group | 2.39 | 6.39 | 0.02 | 0.37 | -10.18 | 14.97 | 0.71 |
| Other ethnic groups | 19.11 | 7.17 | 0.16 | 2.67 | 5.00 | 33.22 | **0.01*** |
| Occupational role |  |  |  |  |  |  |  |
| Medical / Dental | Ref | - | - | - | - | - | - |
| Registered Nurse | -10.51 | 4.69 | -0.22 | -2.24 | -19.74 | -1.28 | **0.03*** |
| Midwives | -6.85 | 5.97 | -0.09 | -1.15 | -18.60 | 4.90 | 0.25 |
| Nursing auxiliary / Nursing assistant / Healthcare assistant | -7.17 | 6.49 | -0.08 | -1.11 | -19.94 | 5.60 | 0.27 |
| Admin and clerical | -9.72 | 6.31 | -0.12 | -1.54 | -22.14 | 2.71 | 0.13 |
| Wider health care team (e.g. Maintenance, Ambulance) | -2.03 | 10.27 | -0.01 | -0.20 | -22.25 | 18.19 | 0.84 |
| Allied health professionals / Healthcare Scientists / Scientific and Technical | -3.65 | 6.20 | -0.04 | -0.59 | -15.85 | 8.56 | 0.56 |
| Presence of long-term health condition |  |  |  |  |  |  |  |
| No | Ref | - | - | - | - | - | - |
| Yes | 5.27 | 2.89 | 0.11 | 1.82 | -0.42 | 10.97 | 0.07 |
| Self-reported sleep duration | -0.61 | 1.23 | -0.03 | -0.50 | -3.04 | 1.81 | 0.62 |
| (Constant) | 43.94 | 12.00 |  | 3.66 | 20.32 | 67.57 | **<0.001*** |

Key: *- Statistically significant difference

S Table 10: Overall work impairment (outcome variable) and probable SWD status (main explanatory variable) Model 2

Adjusted R^2^=0.06, p=0.01, n=311

| **Variables** | **Unstandardized coefficient** | | **Standardized Coefficients β** | **t** | **95% CI** | | **p** |
| --- | --- | --- | --- | --- | --- | --- | --- |
|  | **B** | **Standard error** |  |  | **Lower** | **Upper** |  |
| Probable SWD status | 9.53 | 3.31 | 0.17 | 2.88 | 3.02 | 16.03 | **0.004*** |
| Site |  |  |  |  |  |  |  |
| Site 1 | Ref | - | - | - | - | - | - |
| Site 2 | -3.20 | 3.10 | -0.07 | -1.03 | -9.30 | 2.91 | 0.30 |
| Age | -0.09 | 0.18 | -0.04 | -0.51 | -0.44 | 0.26 | 0.61 |
| Gender |  |  |  |  |  |  |  |
| Male | Ref | - | - | - | - | - | - |
| Female | 3.71 | 3.99 | 0.06 | 0.93 | -4.15 | 11.57 | 0.35 |
| Ethnicity |  |  |  |  |  |  |  |
| White | Ref | - | - | - | - | - | - |
| Asian/Asian British | 0.87 | 3.43 | 0.02 | 0.26 | -5.88 | 7.63 | 0.80 |
| Black / Black British | -3.39 | 4.63 | -0.04 | -0.73 | -12.50 | 5.73 | 0.47 |
| Mixed/Multiple ethnic group | 1.21 | 6.42 | 0.01 | 0.19 | -11.42 | 13.84 | 0.85 |
| Other ethnic groups | 17.39 | 7.24 | 0.14 | 2.40 | 3.15 | 31.63 | **0.02*** |
| Occupational role |  |  |  |  |  |  |  |
| Medical / Dental | Ref | - | - | - | - | - | - |
| Registered Nurse | -10.76 | 4.74 | -0.22 | -2.27 | -20.09 | -1.43 | **0.02*** |
| Midwives | -5.06 | 6.10 | -0.07 | -0.83 | -17.06 | 6.96 | 0.41 |
| Nursing auxiliary / Nursing assistant / Healthcare assistant | -5.93 | 6.57 | -0.07 | -0.90 | -18.87 | 7.00 | 0.37 |
| Admin and clerical | -11.23 | 6.50 | -0.13 | -1.73 | -24.01 | 1.56 | 0.09 |
| Wider health care team (e.g. Maintenance, Ambulance) | -3.27 | 10.44 | -0.02 | -0.31 | -23.82 | 17.27 | 0.75 |
| Allied health professionals / Healthcare Scientists / Scientific and Technical | -5.60 | 6.31 | -0.07 | -0.89 | -18.02 | 6.82 | 0.38 |
| Presence of long-term health condition |  |  |  |  |  |  |  |
| No | Ref | - | - | - | - | - | - |
| Yes | 4.95 | 2.92 | 0.10 | 1.69 | -0.80 | 10.69 | 0.09 |
| Self-reported sleep duration | -0.86 | 1.25 | -0.04 | -0.69 | -3.32 | 1.60 | 0.49 |
| Employment type |  |  |  |  |  |  |  |
| Full-time | Ref | - | - | - | - | - | - |
| Part-time | -3.98 | 3.57 | -0.08 | -1.12 | -11.00 | 3.05 | 0.27 |
| Duration of working shifts (years) | -0.01 | 0.25 | 0.00 | -0.04 | -0.49 | 0.47 | 0.97 |
| Shift type |  |  |  |  |  |  |  |
| Rotating shifts | Ref | - | - | - | - | - | - |
| Evening shift | 2.24 | 4.15 | 0.03 | 0.54 | -5.93 | 10.41 | 0.59 |
| Night shift | -4.82 | 3.72 | -0.08 | -1.30 | -12.14 | 2.49 | 0.20 |
| (Constant) | 46.54 | 12.60 |  | 3.70 | 21.75 | 71.33 | **<0.001*** |

Key: *- Statistically significant difference

S Table 11: Activity impairment (outcome variable) and probable SWD status (main explanatory variable) Model 1

Adjusted R^2^=0.17, p<0.001, n=400

| **Variables** | **Unstandardized coefficient** | | **Standardized Coefficients β** | **t** | **95% CI** | | **p** |
| --- | --- | --- | --- | --- | --- | --- | --- |
|  | **B** | **Standard error** |  |  | **Lower** | **Upper** |  |
| Probable SWD status | 17.43 | 2.81 | 0.30 | 6.20 | 11.90 | 22.95 | **<0.001*** |
| Site |  |  |  |  |  |  |  |
| Site 1 | Ref | - | - | - | - | - | - |
| Site 2 | -2.86 | 2.57 | -0.05 | -1.11 | -7.92 | 2.20 | 0.27 |
| Age | -0.28 | 0.12 | -0.11 | -2.35 | -0.51 | -0.05 | **0.02*** |
| Gender |  |  |  |  |  |  |  |
| Male | Ref | - | - | - | - | - | - |
| Female | 5.77 | 3.50 | 0.08 | 1.65 | -1.11 | 12.65 | 0.10 |
| Ethnicity |  |  |  |  |  |  |  |
| White | Ref | - | - | - | - | - | - |
| Asian/Asian British | -3.96 | 2.96 | -0.07 | -1.34 | -9.78 | 1.85 | 0.18 |
| Black / Black British | -8.91 | 3.82 | -0.12 | -2.33 | -16.42 | -1.41 | **0.02*** |
| Mixed/Multiple ethnic group | 2.04 | 5.80 | 0.02 | 0.35 | -9.38 | 13.45 | 0.73 |
| Other ethnic groups | 12.47 | 6.76 | 0.09 | 1.85 | -0.81 | 25.75 | 0.07 |
| Occupational role |  |  |  |  |  |  |  |
| Medical / Dental | Ref | - | - | - | - | - | - |
| Registered Nurse | -9.07 | 4.43 | -0.17 | -2.05 | -17.78 | -0.37 | **0.04*** |
| Midwives | -2.91 | 5.69 | -0.03 | -0.51 | -14.10 | 8.28 | 0.61 |
| Nursing auxiliary / Nursing assistant / Healthcare assistant | -13.57 | 5.86 | -0.15 | -2.32 | -25.09 | -2.05 | **0.02*** |
| Admin and clerical | -7.15 | 5.48 | -0.09 | -1.31 | -17.92 | 3.62 | 0.19 |
| Wider health care team (e.g. Maintenance, Ambulance) | -4.97 | 9.68 | -0.03 | -0.51 | -24.00 | 14.06 | 0.61 |
| Allied health professionals / Healthcare Scientists / Scientific and Technical | -0.90 | 5.88 | -0.01 | -0.15 | -12.47 | 10.67 | 0.88 |
| Presence of long-term health condition |  |  |  |  |  |  |  |
| No | Ref | - | - | - | - | - | - |
| Yes | 5.15 | 2.58 | 0.10 | 2.00 | 0.08 | 10.23 | 0.05 |
| Self-reported sleep duration | -0.53 | 1.06 | -0.02 | -0.50 | -2.62 | 1.56 | 0.62 |
| (Constant) | 50.98 | 10.47 |  | 4.87 | 30.40 | 71.56 | **<0.001*** |

Key: *- Statistically significant difference

S Table 12: Activity impairment (outcome variable) and probable SWD status (main explanatory variable) Model 2

Adjusted R^2^=0.17, p<0.001, n=400

| **Variables** | **Unstandardized coefficient** | | **Standardized Coefficients β** | **t** | **95% CI** | | **p** |
| --- | --- | --- | --- | --- | --- | --- | --- |
|  | **B** | **Standard error** |  |  | **Lower** | **Upper** |  |
| Probable SWD status | 17.37 | 2.86 | 0.30 | 6.07 | 11.74 | 23.00 | **<0.001*** |
| Site |  |  |  |  |  |  |  |
| Site 1 | Ref | - | - | - | - | - | - |
| Site 2 | -2.57 | 2.78 | -0.05 | -0.92 | -8.03 | 2.90 | 0.36 |
| Age | -0.34 | 0.15 | -0.14 | -2.26 | -0.63 | -0.04 | **0.03*** |
| Gender |  |  |  |  |  |  |  |
| Male | Ref | - | - | - | - | - | - |
| Female | 5.88 | 3.51 | 0.08 | 1.68 | -1.02 | 12.78 | 0.09 |
| Ethnicity |  |  |  |  |  |  |  |
| White | Ref | - | - | - | - | - | - |
| Asian/Asian British | -3.10 | 3.03 | -0.06 | -1.02 | -9.06 | 2.85 | 0.31 |
| Black / Black British | -8.10 | 3.93 | -0.11 | -2.06 | -15.83 | -0.38 | **0.04*** |
| Mixed/Multiple ethnic group | 2.09 | 5.84 | 0.02 | 0.36 | -9.40 | 13.57 | 0.72 |
| Other ethnic groups | 12.52 | 6.82 | 0.09 | 1.83 | -0.90 | 25.93 | 0.07 |
| Occupational role |  |  |  |  |  |  |  |
| Medical / Dental | Ref | - | - | - | - | - | - |
| Registered Nurse | -8.50 | 4.47 | -0.16 | -1.90 | -17.30 | 0.29 | 0.06 |
| Midwives | -1.82 | 5.85 | -0.02 | -0.31 | -13.32 | 9.69 | 0.76 |
| Nursing auxiliary / Nursing assistant / Healthcare assistant | -12.48 | 5.98 | -0.14 | -2.09 | -24.23 | -0.73 | **0.04*** |
| Admin and clerical | -5.41 | 5.63 | -0.07 | -0.96 | -16.47 | 5.67 | 0.34 |
| Wider health care team (e.g. Maintenance, Ambulance) | -3.68 | 9.82 | -0.02 | -0.38 | -22.99 | 15.63 | 0.71 |
| Allied health professionals / Healthcare Scientists / Scientific and Technical | 0.23 | 5.98 | 0.00 | 0.04 | -11.52 | 11.98 | 0.97 |
| Presence of long-term health condition |  |  |  |  |  |  |  |
| No | Ref | - | - | - | - | - | - |
| Yes | 5.54 | 2.62 | 0.10 | 2.12 | 0.39 | 10.68 | **0.04*** |
| Self-reported sleep duration | -0.46 | 1.07 | -0.02 | -0.43 | -2.57 | 1.65 | 0.67 |
| Employment type |  |  |  |  |  |  |  |
| Full-time | Ref | - | - | - | - | - | - |
| Part-time | -2.55 | 3.26 | -0.04 | -0.78 | -8.96 | 3.85 | 0.43 |
| Duration of working shifts (years) | 0.21 | 0.21 | 0.06 | 1.00 | -0.21 | 0.63 | 0.32 |
| Shift type |  |  |  |  |  |  |  |
| Rotating shifts | Ref | - | - | - | - | - | - |
| Evening shift | -3.93 | 3.65 | -0.05 | -1.08 | -11.10 | 3.24 | 0.28 |
| Night shift | 1.65 | 3.27 | 0.03 | 0.51 | -4.78 | 8.08 | 0.61 |
| (Constant) | 50.45 | 10.80 |  | 4.67 | 29.20 | 71.69 | **<0.001*** |

Key: *- Statistically significant difference

S Table 13: Likely depression (outcome variable) and probable SWD status (main explanatory variable) Model 1

Nagelkerke R^2^=0.19, p<0.001, n=400

| **Variables** | **Unstandardized coefficient** | | **Odds Ratio (Exp(B))** | **95% CI** | | **p** |
| --- | --- | --- | --- | --- | --- | --- |
|  | **B** | **Standard error** |  | **Lower** | **Upper** |  |
| Probable SWD status | 1.29 | 0.39 | 3.63 | 1.68 | 7.84 | **0.001*** |
| Site |  |  |  |  |  |  |
| Site 1 | Ref | - | - | - | - | - |
| Site 2 | 0.07 | 0.28 | 1.07 | 0.62 | 1.86 | 0.81 |
| Age | -0.01 | 0.01 | 0.99 | 0.97 | 1.02 | 0.60 |
| Gender |  |  |  |  |  |  |
| Male | Ref | - | - | - | - | - |
| Female | 0.25 | 0.39 | 1.29 | 0.59 | 2.79 | 0.52 |
| Ethnicity |  |  |  |  |  |  |
| White | Ref | - | - | - | - | - |
| Asian/Asian British | 0.42 | 0.33 | 1.53 | 0.80 | 2.91 | 0.20 |
| Black / Black British | 0.41 | 0.42 | 1.51 | 0.66 | 3.42 | 0.33 |
| Mixed/Multiple ethnic group | 0.62 | 0.57 | 1.85 | 0.61 | 5.66 | 0.28 |
| Other ethnic groups | 1.08 | 0.68 | 2.94 | 0.78 | 11.05 | 0.11 |
| Occupational role |  |  |  |  |  |  |
| Medical / Dental | Ref | - | - | - | - | - |
| Registered Nurse | 0.03 | 0.48 | 0.95 | 0.41 | 2.63 | 0.95 |
| Midwives | -0.87 | 0.68 | 0.20 | 0.11 | 1.59 | 0.20 |
| Nursing auxiliary / Nursing assistant / Healthcare assistant | -0.22 | 0.68 | 0.75 | 0.21 | 3.03 | 0.75 |
| Admin and clerical | 0.26 | 0.59 | 0.66 | 0.41 | 4.10 | 0.66 |
| Wider health care team (e.g. Maintenance, Ambulance) | 1.63 | 0.92 | 0.08 | 0.84 | 30.72 | 0.08 |
| Allied health professionals / Healthcare Scientists / Scientific and Technical | -0.23 | 0.65 | 0.73 | 0.22 | 2.86 | 0.73 |
| Presence of long-term health condition |  |  |  |  |  |  |
| No | Ref | - | - | - | - | - |
| Yes | 1.20 | 0.28 | 3.32 | 1.93 | 5.72 | **<0.001*** |
| Self-reported sleep duration | -0.14 | 0.12 | 0.87 | 0.69 | 1.09 | 0.23 |
| (Constant) | -2.28 | 1.20 | 0.10 |  |  | 0.06 |

Key: *- Statistically significant difference

S Table 14: Likely depression (outcome variable) and probable SWD status (main explanatory variable) Model 2

Nagelkerke R^2^=0.22, p<0.001, n=400

| **Variables** | **Unstandardized coefficient** | | **Odds Ratio (Exp(B))** | **95% CI** | | **p** |
| --- | --- | --- | --- | --- | --- | --- |
|  | **B** | **Standard error** |  | **Lower** | **Upper** |  |
| Probable SWD status | 1.47 | 0.41 | 4.35 | 1.97 | 9.61 | **<0.001*** |
| Site |  |  |  |  |  |  |
| Site 1 | Ref | - | - | - | - | - |
| Site 2 | 0.19 | 0.31 | 1.20 | 0.65 | 2.22 | 0.55 |
| Age | 0.01 | 0.02 | 1.01 | 0.98 | 1.05 | 0.41 |
| Gender |  |  |  |  |  |  |
| Male | Ref | - | - | - | - | - |
| Female | 0.27 | 0.40 | 1.31 | 0.60 | 2.88 | 0.50 |
| Ethnicity |  |  |  |  |  |  |
| White | Ref | - | - | - | - | - |
| Asian/Asian British | 0.39 | 0.34 | 1.47 | 0.76 | 2.87 | 0.26 |
| Black / Black British | 0.34 | 0.43 | 1.40 | 0.60 | 3.26 | 0.43 |
| Mixed/Multiple ethnic group | 0.61 | 0.59 | 1.84 | 0.58 | 5.84 | 0.30 |
| Other ethnic groups | 1.04 | 0.69 | 2.84 | 0.74 | 10.95 | 0.13 |
| Occupational role |  |  |  |  |  |  |
| Medical / Dental | Ref | - | - | - | - | - |
| Registered Nurse | 0.16 | 0.49 | 0.74 | 0.45 | 3.07 | 0.74 |
| Midwives | -0.59 | 0.71 | 0.41 | 0.14 | 2.23 | 0.41 |
| Nursing auxiliary / Nursing assistant / Healthcare assistant | -0.14 | 0.70 | 0.84 | 0.22 | 3.46 | 0.84 |
| Admin and clerical | 0.17 | 0.61 | 0.78 | 0.36 | 3.92 | 0.78 |
| Wider health care team (e.g. Maintenance, Ambulance) | 1.46 | 0.96 | 0.13 | 0.66 | 27.98 | 0.13 |
| Allied health professionals / Healthcare Scientists / Scientific and Technical | -0.20 | 0.67 | 0.77 | 0.22 | 3.05 | 0.77 |
| Presence of long-term health condition |  |  |  |  |  |  |
| No | Ref | - | - | - | - | - |
| Yes | 1.31 | 0.29 | 3.71 | 2.11 | 6.51 | **<0.001*** |
| Self-reported sleep duration | -0.09 | 0.12 | 0.91 | 0.73 | 1.15 | 0.44 |
| Employment type |  |  |  |  |  |  |
| Full-time | Ref | - | - | - | - | - |
| Part-time | -0.64 | 0.38 | 0.53 | 0.25 | 1.11 | 0.09 |
| Duration of working shifts (years) | -0.04 | 0.03 | 0.96 | 0.92 | 1.01 | 0.15 |
| Shift type |  |  |  |  |  |  |
| Rotating shifts | Ref | - | - | - | - | - |
| Evening shift | -0.25 | 0.43 | 0.78 | 0.34 | 1.82 | 0.57 |
| Night shift | 0.55 | 0.37 | 1.74 | 0.84 | 3.58 | 0.13 |
| (Constant) | -3.27 | 1.28 | 0.04 |  |  | **0.01*** |

Key: *- Statistically significant difference
S Table 15: Likely anxiety (outcome variable) and probable SWD status (main explanatory variable) Model 1

Nagelkerke R^2^=0.25, p<0.001, n=400

| **Variables** | **Unstandardized coefficient** | | **Odds Ratio (Exp(B))** | **95% CI** | | **p** |
| --- | --- | --- | --- | --- | --- | --- |
|  | **B** | **Standard error** |  | **Lower** | **Upper** |  |
| Probable SWD status | 1.25 | 0.42 | 3.48 | 1.53 | 7.94 | **0.003*** |
| Site |  |  |  |  |  |  |
| Site 1 | Ref | - | - | - | - | - |
| Site 2 | -0.66 | 0.31 | 0.52 | 0.28 | 0.95 | **0.03*** |
| Age | -0.01 | 0.01 | 1.00 | 0.97 | 1.02 | 0.70 |
| Gender |  |  |  |  |  |  |
| Male | Ref | - | - | - | - | - |
| Female | -0.19 | 0.40 | 0.83 | 0.38 | 1.80 | 0.63 |
| Ethnicity |  |  |  |  |  |  |
| White | Ref | - | - | - | - | - |
| Asian/Asian British | 0.05 | 0.35 | 1.05 | 0.53 | 2.09 | 0.88 |
| Black / Black British | -0.60 | 0.49 | 0.55 | 0.21 | 1.43 | 0.22 |
| Mixed/Multiple ethnic group | 0.25 | 0.60 | 1.28 | 0.40 | 4.11 | 0.68 |
| Other ethnic groups | 0.06 | 0.73 | 1.06 | 0.25 | 4.47 | 0.93 |
| Occupational role |  |  |  |  |  |  |
| Medical / Dental | Ref | - | - | - | - | - |
| Registered Nurse | -0.07 | 0.51 | 0.90 | 0.34 | 2.55 | 0.90 |
| Midwives | -0.47 | 0.67 | 0.48 | 0.17 | 2.30 | 0.48 |
| Nursing auxiliary / Nursing assistant / Healthcare assistant | -0.08 | 0.71 | 0.91 | 0.23 | 3.73 | 0.91 |
| Admin and clerical | -0.16 | 0.64 | 0.80 | 0.24 | 2.96 | 0.80 |
| Wider health care team (e.g. Maintenance, Ambulance) | 0.24 | 0.96 | 0.80 | 0.19 | 8.41 | 0.80 |
| Allied health professionals / Healthcare Scientists / Scientific and Technical | 0.10 | 0.67 | 0.88 | 0.30 | 4.09 | 0.88 |
| Presence of long-term health condition |  |  |  |  |  |  |
| No | Ref | - | - | - | - | - |
| Yes | 1.63 | 0.30 | 5.10 | 2.84 | 9.17 | **<0.001*** |
| Self-reported sleep duration | -0.38 | 0.13 | 0.69 | 0.53 | 0.89 | **0.00*** |
| (Constant) | -0.18 | 1.27 | 0.84 |  |  | 0.89 |

Key: *- Statistically significant difference

S Table 16: Likely anxiety (outcome variable) and probable SWD status (main explanatory variable) Model 2

Nagelkerke R^2^=0.26, p<0.001, n=400

| **Variables** | **Unstandardized coefficient** | | **Odds Ratio (Exp(B))** | **95% CI** | | **p** |
| --- | --- | --- | --- | --- | --- | --- |
|  | **B** | **Standard error** |  | **Lower** | **Upper** |  |
| Probable SWD status | 1.26 | 0.43 | 3.52 | 1.52 | 8.12 | **0.003*** |
| Site |  |  |  |  |  |  |
| Site 1 | Ref | - | - | - | - | - |
| Site 2 | -0.51 | 0.34 | 0.60 | 0.31 | 1.17 | 0.13 |
| Age | -0.01 | 0.02 | 0.99 | 0.95 | 1.02 | 0.45 |
| Gender |  |  |  |  |  |  |
| Male | Ref | - | - | - | - | - |
| Female | -0.24 | 0.40 | 0.79 | 0.36 | 1.72 | 0.55 |
| Ethnicity |  |  |  |  |  |  |
| White | Ref | - | - | - | - | - |
| Asian/Asian British | 0.03 | 0.36 | 1.03 | 0.51 | 2.08 | 0.93 |
| Black / Black British | -0.67 | 0.50 | 0.51 | 0.19 | 1.37 | 0.18 |
| Mixed/Multiple ethnic group | 0.21 | 0.60 | 1.23 | 0.38 | 3.99 | 0.73 |
| Other ethnic groups | 0.08 | 0.74 | 1.08 | 0.25 | 4.62 | 0.92 |
| Occupational role |  |  |  |  |  |  |
| Medical / Dental | Ref | - | - | - | - | - |
| Registered Nurse | -0.15 | 0.52 | 0.77 | 0.31 | 2.38 | 0.77 |
| Midwives | -0.44 | 0.70 | 0.53 | 0.16 | 2.53 | 0.53 |
| Nursing auxiliary / Nursing assistant / Healthcare assistant | 0.05 | 0.73 | 0.95 | 0.25 | 4.42 | 0.95 |
| Admin and clerical | -0.23 | 0.66 | 0.73 | 0.22 | 2.90 | 0.73 |
| Wider health care team (e.g. Maintenance, Ambulance) | -0.01 | 0.99 | 0.99 | 0.14 | 6.90 | 0.99 |
| Allied health professionals / Healthcare Scientists / Scientific and Technical | -0.04 | 0.68 | 0.96 | 0.26 | 3.63 | 0.96 |
| Presence of long-term health condition |  |  |  |  |  |  |
| No | Ref | - | - | - | - | - |
| Yes | 1.66 | 0.31 | 5.25 | 2.88 | 9.56 | **<0.001*** |
| Self-reported sleep duration | -0.40 | 0.14 | 0.67 | 0.51 | 0.87 | **0.00*** |
| Employment type |  |  |  |  |  |  |
| Full-time | Ref | - | - | - | - | - |
| Part-time | -0.35 | 0.40 | 0.71 | 0.33 | 1.53 | 0.38 |
| Duration of working shifts (years) | 0.01 | 0.02 | 1.01 | 0.97 | 1.06 | 0.62 |
| Shift type |  |  |  |  |  |  |
| Rotating shifts | Ref | - | - | - | - | - |
| Evening shift | 0.63 | 0.40 | 1.87 | 0.86 | 4.06 | 0.11 |
| Night shift | 0.04 | 0.42 | 1.05 | 0.46 | 2.39 | 0.92 |
| (Constant) | 0.16 | 1.33 | 1.17 |  |  | 0.91 |

Key: *- Statistically significant difference

# Supplementary material 5: Sensitivity Analysis for EQ-5D-5L Utility Scores – Results

Overall, the results of the sensitivity analyses were largely consistent across modelling choices. Probable SWD status, female sex, and the presence of a long-term condition (LTC) were negatively associated with EQ-5D-5L utility scores, while site 2 and self-reported sleep duration were positively associated with utility scores.

Some differences were observed. Age was negatively associated with utility scores in model 2 of the primary analysis; however, this association was not observed in model 1 of the primary analysis or in any sensitivity analysis. The OLS models in both the primary and sensitivity analyses identified statistically significant associations between specific occupational roles (primary analysis: administrative staff and wider healthcare team; sensitivity analysis: nursing assistants, administrative staff, and wider healthcare team) and utility scores. These associations were not evident in the beta regression analyses. In contrast, the beta regression analyses identified a positive association between Asian ethnicity and utility scores in both model 1 and model 2, as well as a positive association between night shift work and utility scores in model 2; these associations were not observed in the OLS models.

S Table 17: OLS analysis with bootstrapping for EQ-5D-5L utility scores (outcome variable) and probable SWD status (main explanatory variable) Model 1 (n=400)

| **Variables** | **Unstandardized coefficient** | | **Bootstrapped**  **95% CI** | | **Statistical significance*** |
| --- | --- | --- | --- | --- | --- |
|  | **B** | **Bootstrapped standard error** | **Lower** | **Upper** |  |
| Probable SWD status | -0.08 | 0.02 | -0.12 | -0.04 | **< 0.05** |
| Age | -0.001 | 0.001 | -0.003 | 0.000 |  |
| Site |  |  |  |  |  |
| Site 1 | Ref | - | - | - | - |
| Site 2 | 0.05 | 0.02 | 0.02 | 0.09 | **< 0.05** |
| Gender |  |  |  |  |  |
| Male | Ref | - | - | - | - |
| Female | -0.05 | 0.02 | -0.09 | 0.00 | **< 0.05** |
| Ethnicity |  |  |  |  |  |
| White | Ref | - | - | - | - |
| Asian/Asian British | 0.01 | 0.02 | -0.03 | 0.05 |  |
| Black / Black British | -0.01 | 0.03 | -0.07 | 0.05 |  |
| Mixed/Multiple ethnic group | -0.03 | 0.04 | -0.12 | 0.05 |  |
| Other ethnic groups | -0.004 | 0.052 | -0.120 | 0.088 |  |
| Occupational role |  |  |  |  |  |
| Medical / Dental | Ref | - | - | - | - |
| Registered Nurse | -0.02 | 0.03 | -0.07 | 0.04 |  |
| Midwives | 0.02 | 0.04 | -0.05 | 0.10 |  |
| Nursing auxiliary / Nursing assistant / Healthcare assistant | -0.08 | 0.04 | -0.14 | -0.01 | **< 0.05** |
| Admin and clerical | -0.11 | 0.04 | -0.20 | -0.03 | **< 0.05** |
| Wider health care team (e.g. Maintenance, Ambulance) | -0.13 | 0.06 | -0.27 | -0.01 | **< 0.05** |
| Allied health professionals / Healthcare Scientists / Scientific and Technical | -0.04 | 0.04 | -0.11 | 0.04 |  |
| Presence of long-term health condition |  |  |  |  |  |
| No | Ref | - | - | - | - |
| Yes | -0.14 | 0.02 | -0.18 | -0.10 | **< 0.05** |
| Self-reported sleep duration | 0.03 | 0.01 | 0.02 | 0.05 | **< 0.05** |
| (Constant) | 0.79 | 0.08 | 0.64 | 0.94 | **< 0.05** |

Key: * - Statistical significance is defined based on whether the 95% CI excludes 0 when bootstrapping is applied. Two decimal places are used unless greater precision is required.

S Table 18: Beta regression analysis with a logit link for EQ-5D-5L utility scores (outcome variable) and probable SWD status (main explanatory variable) Model 1 (n=399)

| **Variables** | **Unstandardized coefficient** | | **95% CI** | | **Statistical significance** |
| --- | --- | --- | --- | --- | --- |
|  | **B** | **Standard error** | **Lower** | **Upper** |  |
| ***Mean model*** |  |  |  |  |  |
| Probable SWD status | -0.41 | 0.11 | -0.62 | -0.20 | **< 0.05** |
| Age | -0.007 | 0.004 | -0.015 | 0.002 |  |
| Site |  |  |  |  |  |
| Site 1 | Ref | - | - | - | - |
| Site 2 | 0.32 | 0.10 | 0.11 | 0.52 | **< 0.05** |
| Gender |  |  |  |  |  |
| Male | Ref | - | - | - | - |
| Female | -0.33 | 0.13 | -0.59 | -0.07 | **< 0.05** |
| Ethnicity |  |  |  |  |  |
| White | Ref | - | - | - | - |
| Asian/Asian British | 0.24 | 0.11 | 0.02 | 0.45 | **< 0.05** |
| Black / Black British | 0.04 | 0.14 | -0.23 | 0.32 |  |
| Mixed/Multiple ethnic group | -0.17 | 0.21 | -0.59 | 0.24 |  |
| Other ethnic groups | 0.21 | 0.25 | -0.28 | 0.70 |  |
| Occupational role |  |  |  |  |  |
| Medical / Dental | Ref | - | - | - | - |
| Registered Nurse | -0.15 | 0.17 | -0.48 | 0.18 |  |
| Midwives | 0.21 | 0.22 | -0.22 | 0.63 |  |
| Nursing auxiliary / Nursing assistant / Healthcare assistant | -0.35 | 0.21 | -0.77 | 0.06 | < 0.1 |
| Admin and clerical | -0.38 | 0.21 | -0.79 | 0.02 | < 0.1 |
| Wider health care team (e.g. Maintenance, Ambulance) | -0.67 | 0.35 | -1.35 | 0.01 | < 0.1 |
| Allied health professionals / Healthcare Scientists / Scientific and Technical | -0.17 | 0.22 | -0.60 | 0.26 |  |
| Presence of long-term health condition |  |  |  |  |  |
| No | Ref | - | - | - | - |
| Yes | -0.81 | 0.10 | -1.02 | -0.61 | **< 0.05** |
| Sleep duration | 0.16 | 0.04 | 0.09 | 0.24 | **< 0.05** |
| (Constant) | 1.45 | 0.39 | -0.69 | 2.21 | **< 0.05** |
| ***Precision model*** |  |  |  |  |  |
| Site |  |  |  |  |  |
| Site 1 | Ref | - | - | - | - |
| Site 2 | 0.29 | 0.14 | 0.02 | 0.56 | **< 0.05** |
| Presence of long-term health condition |  |  |  |  |  |
| No | Ref | - | - | - | - |
| Yes | -0.62 | 0.14 | -0.90 | -0.35 | **< 0.05** |
| (Constant) | 1.85 | 0.11 | 1.63 | 2.06 | **< 0.05** |

Key: * - Results are reported on the log-odds (logit) scale, consistent with the logit link function used in the beta regression. One observation (n = 1; 0.25%) with a negative utility value was excluded as this observation lay outside the theoretical support of the beta distribution (0,1). The analysis therefore included 399 participants.

S Table 19: OLS analysis with bootstrapping for EQ-5D-5L utility scores (outcome variable) and probable SWD status (main explanatory variable) Model 2 (n=400)

| **Variables** | **Unstandardized coefficient** | | **Bootstrapped**  **95% CI** | | **Statistical significance*** |
| --- | --- | --- | --- | --- | --- |
|  | **B** | **Bootstrapped standard error** | **Lower** | **Upper** |  |
| Probable SWD status | -0.08 | 0.02 | -0.12 | -0.04 | **< 0.05** |
| Age | -0.002 | 0.001 | -0.005 | 0.000 |  |
| Site |  |  |  |  |  |
| Site 1 | Ref | - | - | - | - |
| Site 2 | 0.05 | 0.02 | 0.01 | 0.09 | **< 0.05** |
| Gender |  |  |  |  |  |
| Male | Ref | - | - | - | - |
| Female | -0.05 | 0.02 | -0.10 | -0.01 | **< 0.05** |
| Ethnicity |  |  |  |  |  |
| White | Ref | - | - | - | - |
| Asian/Asian British | 0.02 | 0.02 | -0.03 | 0.05 |  |
| Black / Black British | -0.004 | 0.030 | -0.065 | 0.056 |  |
| Mixed/Multiple ethnic group | -0.02 | 0.04 | -0.11 | 0.06 |  |
| Other ethnic groups | 0.01 | 0.05 | -0.10 | 0.10 |  |
| Occupational role |  |  |  |  |  |
| Medical / Dental | Ref | - | - | - | - |
| Registered Nurse | -0.02 | 0.03 | -0.07 | 0.04 |  |
| Midwives | 0.01 | 0.04 | -0.06 | 0.09 |  |
| Nursing auxiliary / Nursing assistant / Healthcare assistant | -0.08 | 0.04 | -0.15 | -0.01 | **< 0.05** |
| Admin and clerical | -0.11 | 0.04 | -0.19 | -0.02 | **< 0.05** |
| Wider health care team (e.g. Maintenance, Ambulance) | -0.13 | 0.07 | -0.27 | -0.02 | **< 0.05** |
| Allied health professionals / Healthcare Scientists / Scientific and Technical | -0.03 | 0.04 | -0.11 | 0.04 |  |
| Presence of long-term health condition |  |  |  |  |  |
| No | Ref | - | - | - | - |
| Yes | -0.14 | 0.02 | -0.17 | -0.10 | **< 0.05** |
| Sleep duration | 0.03 | 0.01 | 0.02 | 0.05 | **< 0.05** |
| Employment Status |  |  |  |  |  |
| Full-time | Ref | - | - | - | - |
| Part-time | 0.004 | 0.023 | -0.041 | 0.050 |  |
| Shift work duration | 0.002 | 0.002 | -0.001 | 0.005 |  |
| Shift work type |  |  |  |  |  |
| Rotating | Ref | - | - | - | - |
| Evening | 0.03 | 0.02 | -0.01 | 0.08 |  |
| Night | 0.03 | 0.02 | -0.01 | 0.08 |  |
| (Constant) | 0.80 | 0.08 | 0.64 | 0.95 | **< 0.05** |

Key: * - Statistical significance is defined based on whether the 95% CI excludes 0 when bootstrapping is applied. Two decimal places are used unless greater precision is required.

S Table 20: Beta regression analysis with a logit link for EQ-5D-5L utility scores (outcome variable) and probable SWD status (main explanatory variable) Model 2 (n=399)

| **Variables** | **Unstandardized coefficient** | | **95% CI** | | **Statistical significance** |
| --- | --- | --- | --- | --- | --- |
|  | **B** | **Standard error** | **Lower** | **Upper** |  |
| ***Mean model*** |  |  |  |  |  |
| Probable SWD status | -0.39 | 0.11 | -0.60 | -0.18 | **< 0.05** |
| Age | -0.01 | 0.01 | 0.02 | 0.00 | < 0.1 |
| Site |  |  |  |  |  |
| Site 1 | Ref | - | - | - | - |
| Site 2 | 0.26 | 0.11 | 0.04 | 0.48 | **< 0.05** |
| Gender |  |  |  |  |  |
| Male | Ref | - | - | - | - |
| Female | -0.32 | 0.13 | -0.58 | -0.06 | **< 0.05** |
| Ethnicity |  |  |  |  |  |
| White | Ref | - | - | - | - |
| Asian/Asian British | 0.26 | 0.11 | 0.05 | 0.48 | **< 0.05** |
| Black / Black British | 0.10 | 0.14 | -0.19 | 0.38 |  |
| Mixed/Multiple ethnic group | -0.15 | 0.21 | -0.55 | 0.26 |  |
| Other ethnic groups | 0.26 | 0.25 | -0.24 | 0.75 |  |
| Occupational role |  |  |  |  |  |
| Medical / Dental | Ref | - | - | - | - |
| Registered Nurse | -0.18 | 0.17 | -0.50 | 0.15 |  |
| Midwives | 0.20 | 0.22 | -0.23 | 0.62 |  |
| Nursing auxiliary / Nursing assistant / Healthcare assistant | -0.39 | 0.21 | -0.80 | 0.03 | < 0.1 |
| Admin and clerical | -0.30 | 0.21 | -0.71 | 0.12 |  |
| Wider health care team (e.g. Maintenance, Ambulance) | -0.64 | 0.36 | -1.35 | 0.06 | < 0.1 |
| Allied health professionals / Healthcare Scientists / Scientific and Technical | -0.14 | 0.22 | -0.58 | 0.31 |  |
| Presence of long-term health condition |  |  |  |  |  |
| No | Ref | - | - | - | - |
| Yes | -0.80 | 0.10 | -0.99 | -0.60 | **< 0.05** |
| Sleep duration | 0.16 | 0.04 | 0.08 | 0.24 | **< 0.05** |
| Employment Status |  |  |  |  |  |
| Full-time | Ref | - | - | - | - |
| Part-time | 0.04 | 0.12 | -0.20 | 0.29 |  |
| Shift work duration | 0.01 | 0.01 | -0.01 | 0.02 |  |
| Shift work type |  |  |  |  |  |
| Rotating | Ref | - | - | - | - |
| Evening | 0.18 | 0.13 | -0.08 | 0.44 |  |
| Night | 0.34 | 0.12 | 0.11 | 0.57 | **< 0.05** |
| (Constant) | 1.37 | 0.40 | 0.60 | 2.15 | **< 0.05** |
| ***Precision model*** |  |  |  |  |  |
| Site |  |  |  |  |  |
| Site 1 | Ref | - | - | - | - |
| Site 2 | 0.22 | 0.15 | -0.07 | 0.51 |  |
| Presence of long-term health condition |  |  |  |  |  |
| No | Ref | - | - | - | - |
| Yes | -0.67 | 0.14 | -0.94 | -0.40 | **< 0.05** |
| Employment Status |  |  |  |  |  |
| Full-time | Ref | - | - | - | - |
| Part-time | 0.38 | 0.17 | 0.04 | 0.71 | **< 0.05** |
| Shift work duration | 0.01 | 0.01 | -0.01 | 0.03 |  |
| (Constant) | 1.72 | 0.13 | 1.47 | 1.98 | **< 0.05** |

Key: * - Results are reported on the log-odds (logit) scale, consistent with the logit link function used in the beta regression. One observation (n = 1; 0.25%) with a negative utility value was excluded as this observation lay outside the theoretical support of the beta distribution (0,1). The analysis therefore included 399 participants.
